# Bacterial pathogens deliver water- and solute-permeable channels to plant cells

**DOI:** 10.1038/s41586-023-06531-5

**Published:** 2023-09-13

**Authors:** Kinya Nomura, Felipe Andreazza, Jie Cheng, Ke Dong, Pei Zhou, Sheng Yang He

**Affiliations:** 1 Department of Biology, Duke University, Durham, NC USA; 2 Howard Hughes Medical Institute, Duke University, Durham, NC USA; 3 Department of Biochemistry, Duke University School of Medicine, Durham, NC USA

**Keywords:** Bacterial pathogenesis, Effectors in plant pathology

## Abstract

Many animal- and plant-pathogenic bacteria use a type III secretion system to deliver effector proteins into host cells^1,2^. Elucidation of how these effector proteins function in host cells is critical for understanding infectious diseases in animals and plants^3–5^. The widely conserved AvrE-family effectors, including DspE in *Erwinia amylovora* and AvrE in *Pseudomonas syringae*, have a central role in the pathogenesis of diverse phytopathogenic bacteria^6^. These conserved effectors are involved in the induction of ‘water soaking’ and host cell death that are conducive to bacterial multiplication in infected tissues. However, the exact biochemical functions of AvrE-family effectors have been recalcitrant to mechanistic understanding for three decades. Here we show that AvrE-family effectors fold into a β-barrel structure that resembles bacterial porins. Expression of AvrE and DspE in *Xenopus* oocytes results in inward and outward currents, permeability to water and osmolarity-dependent oocyte swelling and bursting. Liposome reconstitution confirmed that the DspE channel alone is sufficient to allow the passage of small molecules such as fluorescein dye. Targeted screening of chemical blockers based on the predicted pore size (15–20 Å) of the DspE channel identified polyamidoamine dendrimers as inhibitors of the DspE/AvrE channels. Notably, polyamidoamines broadly inhibit AvrE and DspE virulence activities in *Xenopus* oocytes and during *E. amylovora* and *P. syringae* infections. Thus, we have unravelled the biochemical function of a centrally important family of bacterial effectors with broad conceptual and practical implications in the study of bacterial pathogenesis.

## Abstract (web summary)

The bacterial effector proteins of the AvrE family function as membrane channels in plant cells, enabling the passage of water and solutes from host cells to support pathogen growth, and disrupting the viability of the host cell.

## Main

All AvrE/DspE-family effectors examined, including AvrE from *P. syringae*, WtsE from *Pantoea stewartii*, DspA/E (DspE hereinafter) from *E.* *amylovora* and DspE from *Pectobacterium carotovorum*, are major virulence factors responsible for bacterial multiplication and induction of major disease symptoms including water soaking and host cell death during infection^7–19^. AvrE-family effectors have been challenging to study owing to their extremely large size (approximately 200 kDa), high toxicity to plant and yeast cells and the fact that they share few sequence similarities with proteins of known function^20,21^. Several AvrE-family effectors were reported to interact with plant proteins, including plant protein phosphatase PP2A subunits, type one protein phosphatases and receptor-like kinases^22–25^. In addition, a yeast *cdc55* mutation affecting Cdc55-PP2A protein phosphatase activity was found to suppress DspE-induced yeast growth arrest^21^. Although these interactions associate AvrE-family effectors with various host cellular processes, the fundamental question regarding the actual biochemical function of AvrE-family effectors has remained elusive.

In this study, we carried out AlphaFold2 analysis of the three-dimensional models of AvrE-family proteins. Unexpectedly, AlphaFold2 predicts that this family of proteins fold into a porin-like β-barrel structure. This prediction prompted us to conduct a series of cryogenic electron microscopy (cryo-EM) imaging, *Xenopus* oocyte, liposome and in planta experiments. Our results show that AvrE-family effectors are water- and solute-permeable channels that can be blocked by polyamidoamine (PAMAM) dendrimers. The discovery of the water- and solute-permeable channel function of AvrE/DspE-family effectors solves a decades-long puzzle regarding one of the most important families of phytobacterial type III effectors and marks a major advance in understanding bacterial pathogenesis.

## AlphaFold2 analysis and cryo-EM imaging

**Fig. 1:**
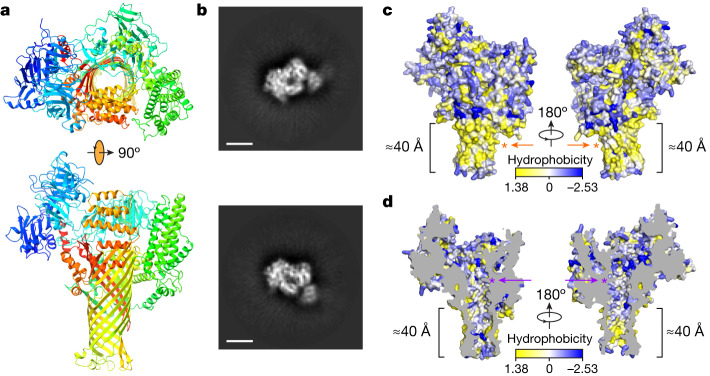
Model and cryo-EM images of *E.* *amylovora* DspE. **a**, Three-dimensional model of *E.* *amylovora* DspE generated by AlphaFold2 using MMseqs2 (ColabFold). DspE (residues 298–1838) is shown in the cartoon model in a rainbow colour gradient, with the N terminus in blue and the C terminus in red. **b**, Cryo-EM two-dimensional class averages of DspE, revealing a circular arrangement of domains around a pore. Scale bars, 5 nm. The result is representative of three experimental replicates. **c**, Surface representation of DspE. **d**, Sliced view of DspE. In **c**,**d**, residues are coloured on the basis of their hydrophobicity scale. The length of the proposed membrane-spanning β-barrel stem is labelled. Orange and purple asterisks mark the approximate locations of the hydrophobic cluster of L1776, L1777 and L1778 and the basic cluster of K1399 and K1401, respectively.

**Extended Data Fig. 1:**
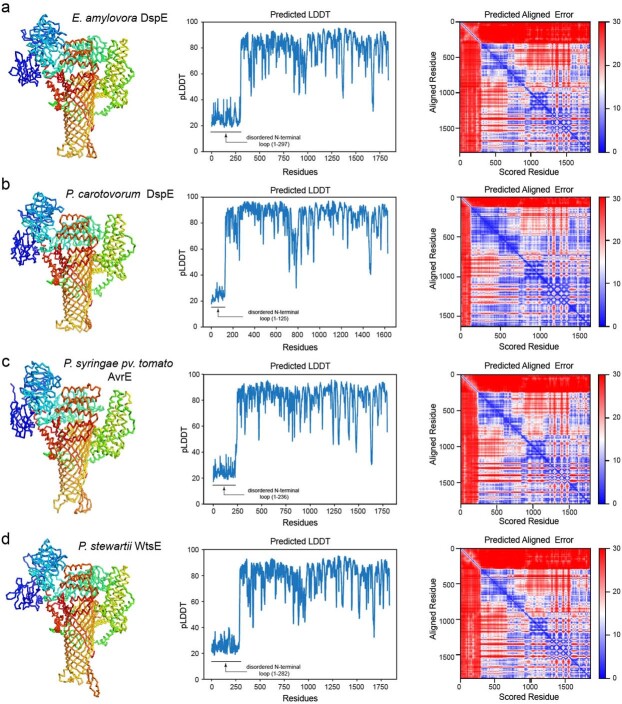
AlphaFold2 models of the AvrE-family effector proteins. The top-ranked models, predicted local distance difference test (pLDDT), and predicted aligned error (PAE) of *E. amylovora* DspE, *P. carotovorum* DspE, *P. syringae* pv. tomato AvrE, and *P. stewartii* WtsE are shown in panels **a**, **b**, **c**, and **d**, respectively. The models are predicted by AlphaFold2 using MMseqs2 (ColabFold). For each protein, the N-terminal disordered loop with low pLDDT scores is not shown, while the remaining residues are shown in Cα traces and colored in rainbow, with the N-terminus in blue and C-terminus in red.

**Extended Data Fig. 2:**
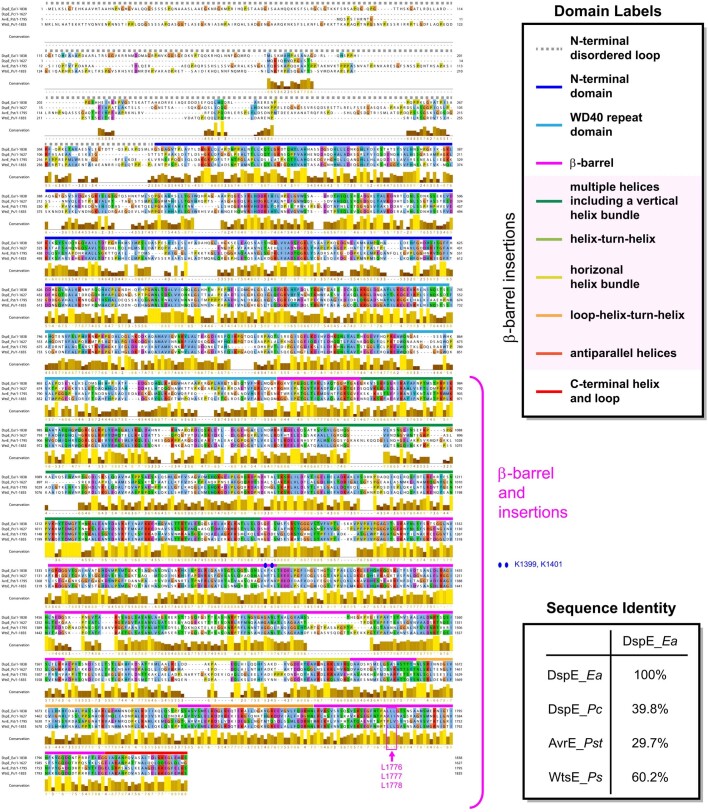
Sequence alignment of representative AvrE-family effectors. Sequences of *E. amylovora* DspE, *P. carotovorum* DspE, *Pst* DC3000 AvrE, and *P. stewartii* WtsE are aligned using Clustal Omega^44^. Domain regions are indicated above the aligned sequences. Sequence identities between different protein pairs are labeled. The β-barrel is formed with multiple helices and long loops inserted within the primary sequence of the β-barrel. In *E. amylovora* DspE, the β-barrel starts from a small β-hairpin from K932-E956, which is followed by a large insertion of multiple helices (including a vertical helix bundle) from E957-D1276. The main β-barrel follows from S1277-G1813, though it is disrupted by several insertion loops and helices, including T1403-E1430 (a helix-turn-helix motif), A1567-H1647 (a horizontal helix bundle), N1662-P1712 (an insertion loop followed by a helix-turn-helix motif), and K1723-N1753 (two antiparallel helices). The β-barrel is further appended with a C-terminal helix and an extended loop (G1814-S1838). Mutated outward-facing residues (L1776, L1777, and L1778) and inward-facing residues (K1399 and K1401) of the β-barrel are labeled in pink and blue, respectively. Source data

To gain functional insights into the AvrE family of bacterial effectors, we constructed their three-dimensional models predicted by AlphaFold2^26^ using the fast homology search of MMseqs2 (ColabFold)^27^. The predicted AlphaFold2 models of DspE from *E.* *amylovora*, DspE from *P.* *carotovorum*, AvrE from *P.* *syringae* pv. *tomato* (*Pst*) DC3000 and WtsE from *P.* *stewartii* (Fig. 1 and Extended Data Figs. 1 and 2) all reveal an overall similar architecture resembling a mushroom, with a prominent central β-barrel forming the stem, which is surrounded by a globular amino-terminal domain (*E.* *amylovora* DspE: K298–H672), a WD40 repeat domain (H673–P912) and two perpendicularly arranged helix bundles (E998–T1222 and A1567–H1647) on the top. The predicted domain arrangement is supported by our cryo-EM imaging of *E.* *amylovora* DspE, for which the two-dimensional class averages clearly reveal an overall similar top view to that of the AlphaFold model, with circularly arranged globular domains surrounding a central pore (Fig. 1a,b).

Further examination of the predicted *E.* *amylovora* DspE three-dimensional model reveals that surface β-barrel residues facing outside are enriched with hydrophobic amino acids (Fig. 1c), whereas inward-facing pore residues are predominantly hydrophilic (Fig. 1d). The length of the lower portion of the β-barrel stem covered by hydrophobic residues is estimated to be about 40 Å, roughly the thickness of a cellular membrane. As AvrE has previously been reported to be membrane anchored^20^, the β-barrel stem of AvrE-family effectors probably inserts into the membrane and functions as a channel, similar to that of bacterial porins^28^. Such a mode of insertion is distinct from that of pore-forming bacterial toxins, such as staphylococcal α-haemolysin and *Clostridium perfringens* β-toxin, for which the β-barrel is assembled through oligomerization of two long β-strands^29,30^.

## DspE and AvrE currents in *Xenopus* oocytes

**Fig. 2:**
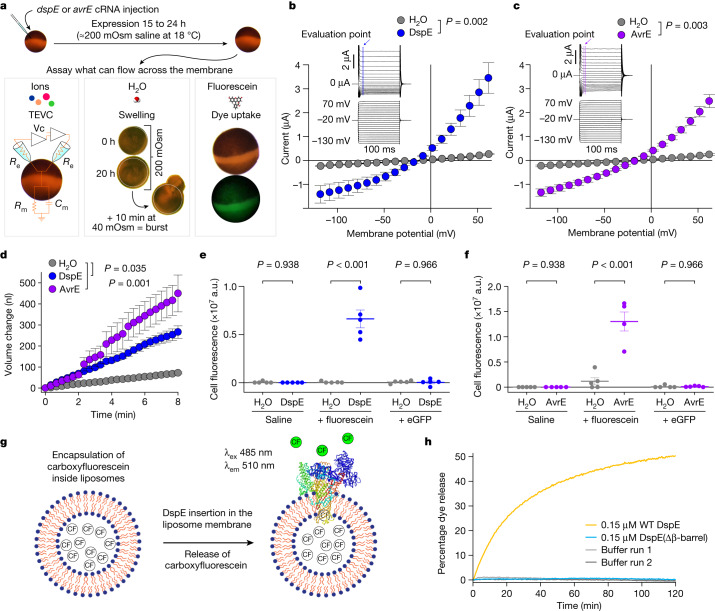
DspE and AvrE activities in *Xenopus* oocytes and liposome. **a**, Schematic of the three oocyte assays, showing details for the two-electrode voltage clamp (TEVC) to test ion conductance (bottom left) (Vc, voltage command; *R*_e_, electrode resistance; *R*_m_, membrane resistance; and *C*_m_, membrane capacitance), the baseline and induced swelling and burst assay to test water conductance (bottom middle) and dye uptake to test conductance to molecules larger than single ions (bottom right). **b**,**c**, DspE and AvrE induce ion currents in the two-electrode voltage clamp assay. Mean ± s.e.m. (*n* = 5 oocytes) current values at different test pulses from oocytes expressing DspE (0.01 ng cRNA per oocyte) or AvrE (0.1 ng cRNA per oocyte) were recorded. **d**, DspE (2 ng) and AvrE (20 ng) induced fast oocyte swelling and burst at 24 h after cRNA injection when placed in a low-osmolarity (40 mOsm) solution. The data are presented as mean ± s.e.m. (*n* = 5 oocytes) of increased oocyte volume in relation to its initial volume. **e**,**f**, Fluorescein or eGFP entry assays. Oocytes injected with 2 ng *dspE* (**e**) or 20 ng *avrE* (**f**) cRNA or injected with water were incubated for 20 h in bath saline with or without fluorescein or eGFP. Values of fluorescence intensity were subtracted from the background and are presented as mean ± s.e.m. (*n* = 5 oocytes) corrected ‘total cell fluorescence’. a.u., arbitrary units. **g**, Schematic of DspE-dependent release of carboxyfluorescein (CF) encapsulated within a liposome. **h**, Fluorescence increased over time for carboxyfluorescein-loaded liposome after addition of wild-type (WT) DspE (yellow), DspE(Δβ-barrel) (blue) or buffer (grey). The result is representative of three experimental replicates. *P* values were calculated using two-way analysis of variance (ANOVA; **b**,**c**,**e**,**f**) or two-way repeated measures ANOVA (**d**). Source data

**Extended Data Fig. 3:**
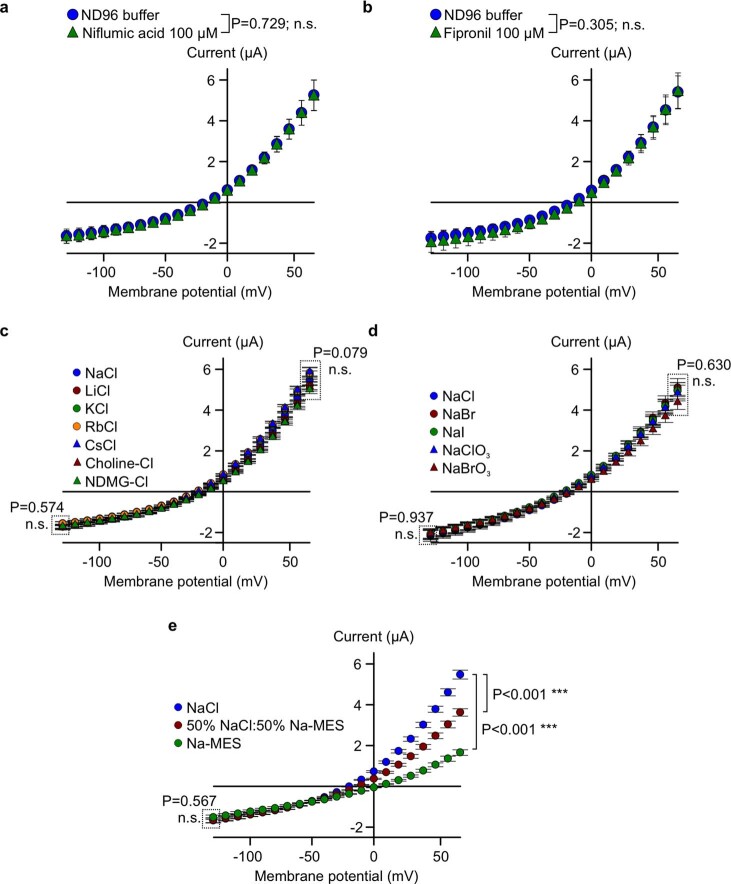
Characterization of DspE-induced whole-cell currents. **a**,**b**, DspE-induced currents were not inhibited by niflumic acid or fipronil. Current values (mean ± s.e.m.; n = 5 oocytes) at different test pulses from oocytes expressing DspE proteins were recorded once in the ND96 recording buffer at 15 h after injection with 0.01 ng cRNA, and a second time after 10 min of incubation with 100 µM of each inhibitor. **c**, Cation replacement experiment. After 15 h of incubation, cells were recorded in the normal ND96 buffer, and then in a new recording solution where sodium in ND96 was replaced with another cation (see details in the Methods section). The data show mean ± s.e.m. (n = 5 oocytes) values at each test pulse for each cation after subtracting currents from control cells. **d**,**e**, Anion replacement experiment. Same as presented in c (mean ± s.e.m., n = 5 oocytes), but with chloride in ND96 being replaced by other elementary anions (d) the organic anion MES^–^ (e). In **e**, either 100% or only 50% of the Cl^–^ was replaced by MES^–^. Experiments were independently performed three (a,c,e) or two (b,d) times with similar results. Two-way ANOVA values and exact P-values for all comparisons are detailed in the Source Data files. Source data

**Extended Data Fig. 4:**
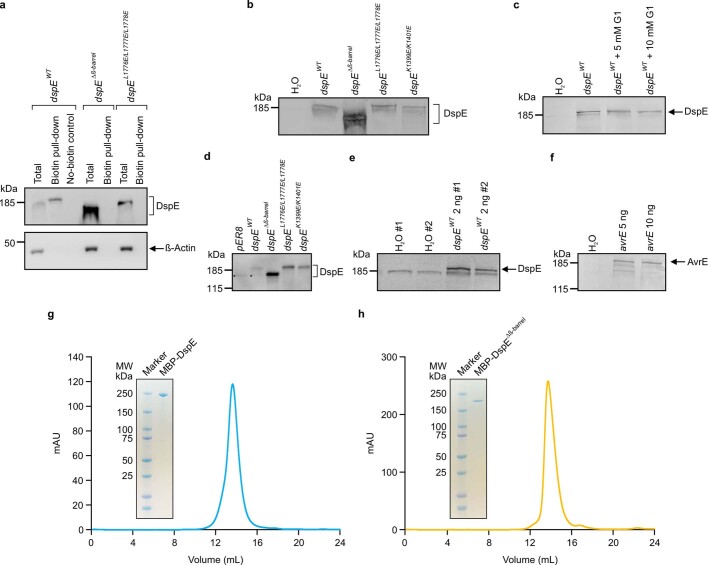
Immunoblot detection of AvrE and DspE proteins and elution profile of MBP-DspE and MBP-DspE^Δβ-barrel^ proteins. **a**, Oocyte surface biotinylation assay of DspE. Two nanograms of wild-type or mutant *dspE* cRNA was injected into oocytes, which were incubated in ND96 media at 18 °C for 15 h before being subjected to surface protein biotinylation assay (see Methods). Anti-β-Actin antibody detect β-Actin, a cytoplasmic protein serving as a negative control. **b**,**c**,**e**,**f**, Detection of AvrE and DspE proteins expressed in oocytes. Oocytes injected with 1 ng (unless otherwise noted in the gel lane label) *avrE*, *dspE* or mutant *dspE* cRNA or H_2_O (control) were incubated in ND96 media at 18 °C for 19 h before being subjected to SDS-PAGE and immunoblot analysis. In **c**, oocytes were incubated in the absence or presence of 5 or 10 mM PAMAM G1. **d**, Detection of DspE proteins expressed in tobacco leaves. 1 × 10^8^ CFU/mL of *Agrobacterium tumefaciens* GV3101 containing pER8 empty vector, pER-*dspE* or pER-*dspE* mutants were syringe-inoculated into leaves of *Nicotiana benthamiana*, kept at 22 °C for 24 h before leaves were painted with 90 μM estradiol to induce protein expression for 8 h and then subjected to SDS-PAGE and immunoblotting. Asterisks in the pER8 and DspE^WT^ lanes indicate a faint nonspecific protein band. Because active DspE expressed much more poorly in plant cells than mutant DspE proteins, mutant DspE samples were diluted 20 times with 2×SDS sample buffer (See Supplementary Fig. 1d). Experiments were independently performed two times. See Supplementary Fig. 1 for image cropping. **g**,**h**, Purified WT MBP-DspE (g) or MBP-DspE^Δβ-barrel^ (h) was eluted on a Suprose 6 increase 10/300 GL column and was analyzed on a SDS-PAGE gel. FPLC traces and gel images in g and h are representative of 3 experimental replicates. See Supplementary Information Fig. 1 for image cropping. Source data

The prediction that AvrE-family effectors are channel-forming proteins prompted us to conduct experiments to test the hypothesis that AvrE and DspE may allow ion conductance when expressed in *Xenopus* oocytes. As shown in the current–voltage relationship (Fig. 2b,c), inward currents and outward currents at negative and positive test potentials, respectively, were detected from oocytes injected with *dspE* or *avrE* complementary RNA (cRNA). The reversal potentials for DspE and AvrE channels are approximately −25 mV. The currents were not affected by niflumic acid, which blocks an endogenous Ca^2+^-activated Cl^−^ channels^31^, or fipronil, which inhibits GABA-gated Cl^−^ channels and glutamate-gated Cl^−^ channels^32^ (Extended Data Fig. 3a,b). Surface biotinylation experiment with oocytes expressing DspE confirmed that this protein is anchored across the oocyte membrane (Extended Data Fig. 4a).

We further characterized whole-cell currents from DspE-expressing oocytes by conducting ion permeability experiments. Replacing extracellular sodium in ND96 recording solution (Methods) with potassium or other cations caused only minor variations in the magnitude of DspE currents (Extended Data Fig. 3c). Similarly, only minor variations in the magnitude of DspE currents were observed when extracellular Cl^−^ was replaced with various anions, except for 4-morpholineethanesulfonic acid, sodium salt (Na-MES; Extended Data Fig. 3d,e). When 50% or 100% of the NaCl was replaced by Na-MES, progressively smaller outward currents were observed, and the reversal potential was shifted to a less negative value (Extended Data Fig. 3e). However, the negative reversal potential was not affected by replacement of other ions. These results suggest that Cl^−^ may have a major role in carrying the outward current and that the DspE channel seems to have some selectivity towards anions including Cl^−^. Future research is needed to comprehensively survey possible ion selectivity of these channels.

## DspE and AvrE induce cell swelling

**Extended Data Fig. 5:**
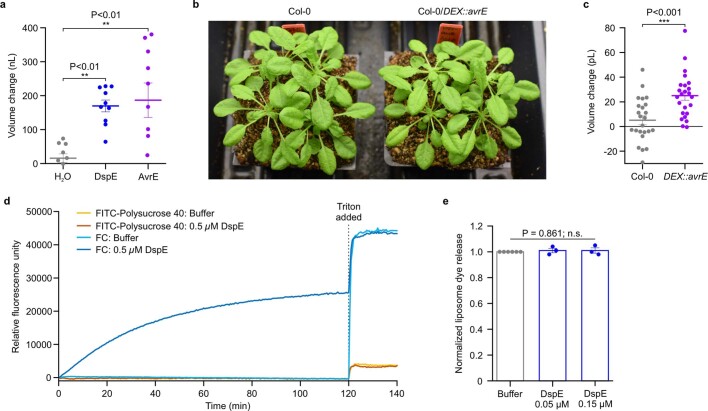
DspE activities in *Xenopus* oocytes, Arabidopsis and liposome. **a**, DspE and AvrE induced a baseline swelling of many oocytes in 200 mOsm bath saline. Oocytes were imaged and measured at 24 h after 2 ng *dspE* or 20 ng *avrE* cRNA injection. Plot shows mean ± s.e.m. (n = 10 oocytes) and individual replicate values for the increased oocyte volume in relation to its initial volume. See Extended Data Fig. 4e, f for immunoblotting of DspE and AvrE proteins expressing in oocytes. **b**, Five-week-old Arabidopsis wild type Col-0 and transgenic Col-0/*DEX::his-avrE* plants (basal expression without dexamethazone induction). **c**, Changes in protoplast volume (mean ± s.e.m.; n = 24 protoplasts) when isolated protoplasts were incubated in protoplast incubation buffer containing 320 mM (low osmolarity) mannitol for 1 h compared to protoplast incubation buffer containing normal 400 mM mannitol for 1 h before image capture and volume analysis with Image J software. **d**, Liposome dye release assay using fluorescein isothiocyanate conjugated polysucrose 40 (FITC-polysucrose 40) and carboxyfluorescein (CF). **e**, Normalized liposome dye release (mean ± s.e.m.; n = 3 batches of liposome preparations) after addition of triton. Raw fluorescence readings were normalized to the buffer control samples. Experiments presented in this figure were independently performed two times. One-way ANOVA on Ranks (a,e) or two-tailed student’s *t*-test (c) values and exact P-values for all comparisons are detailed in the Source Data files. Source data

During voltage-clamp current recording experiments, we noticed that many oocytes injected with *dspE* or *avrE* cRNA showed baseline swelling (Extended Data Fig. 5a), reminiscent of oocytes expressing plant aquaporins^33^. This raised the possibility that AvrE and DspE proteins may function like aquaporin channels to allow water to pass through cell membranes along an osmotic gradient, assuming that the osmolarity of the oocyte bathing medium (about 200 mOsm) may be lower than that of the oocyte cytoplasm. To more directly test this possibility, we adopted an oocyte swelling assay used for aquaporins and transferred oocytes from 200 mOsm to 40 mOsm bathing medium to create a larger osmotic difference. Notably, both DspE- and AvrE-expressing oocytes markedly swelled (Fig. 2d) and eventually burst (Supplementary Videos 1 and 2). We conducted further experiments to determine whether plant cells expressing AvrE would swell using our previously produced transgenic *DEX::avrE* plants^20^. As shown in Extended Data Fig. 5b,c, *Arabidopsis* leaf protoplasts expressing AvrE swelled to a greater extent compared to control *Arabidopsis* leaf protoplasts that have endogenous aquaporins. This provides further evidence that the AvrE channel has an ability to increase water permeability in planta^7–19^.

## DspE channel size selectivity

The predicted AvrE/DspE channels have a diameter of 15–20 Å, much larger than the size of a water molecule or a simple ion. We reasoned that, in addition to ions and water, the AvrE and DspE channels may allow larger molecules to pass through. We conducted fluorescent dye permeability assays to test whether molecules smaller than the predicted pore size could pass through the membrane, whereas molecules larger than the predicted pore size could not. Two fluorescent molecules were tested: fluorescein (molecular mass of 332 Da with an estimated maximum molecular diameter of 7 Å) and enhanced green fluorescent protein (eGFP; molecular mass of 27 kDa with an estimated minimum diameter of 30 Å). As shown in Fig. 2e,f, fluorescein entered oocytes expressing DspE or AvrE, whereas eGFP could not, consistent with the predicted AvrE/DspE channel diameter. Notably, liposome-based in vitro reconstitution of DspE was sufficient to cause time-dependent release of carboxyfluorescein (molecular mass of 376 Da) encapsulated within soybean liposomes, whereas neither the buffer control nor the DspE(Δβ-barrel) mutant, in which most β-barrel-forming sequences are deleted, exhibited substantial activity (Fig. 2g,h), suggesting that no other protein is needed for baseline passage of small molecules through the DspE channel. As in the oocyte experiment, although DspE readily induced carboxyfluorescein dye release from liposomes (Fig. 2h), no time-dependent release of large molecules, such as fluorescein isothiocyanate–polysucrose 40 (molecular mass of 30–50 kDa with an estimated diameter of 80 Å), was observed (Extended Data Fig. 5d).

## Mutational analysis of the DspE channel

**Extended Data Fig. 6:**
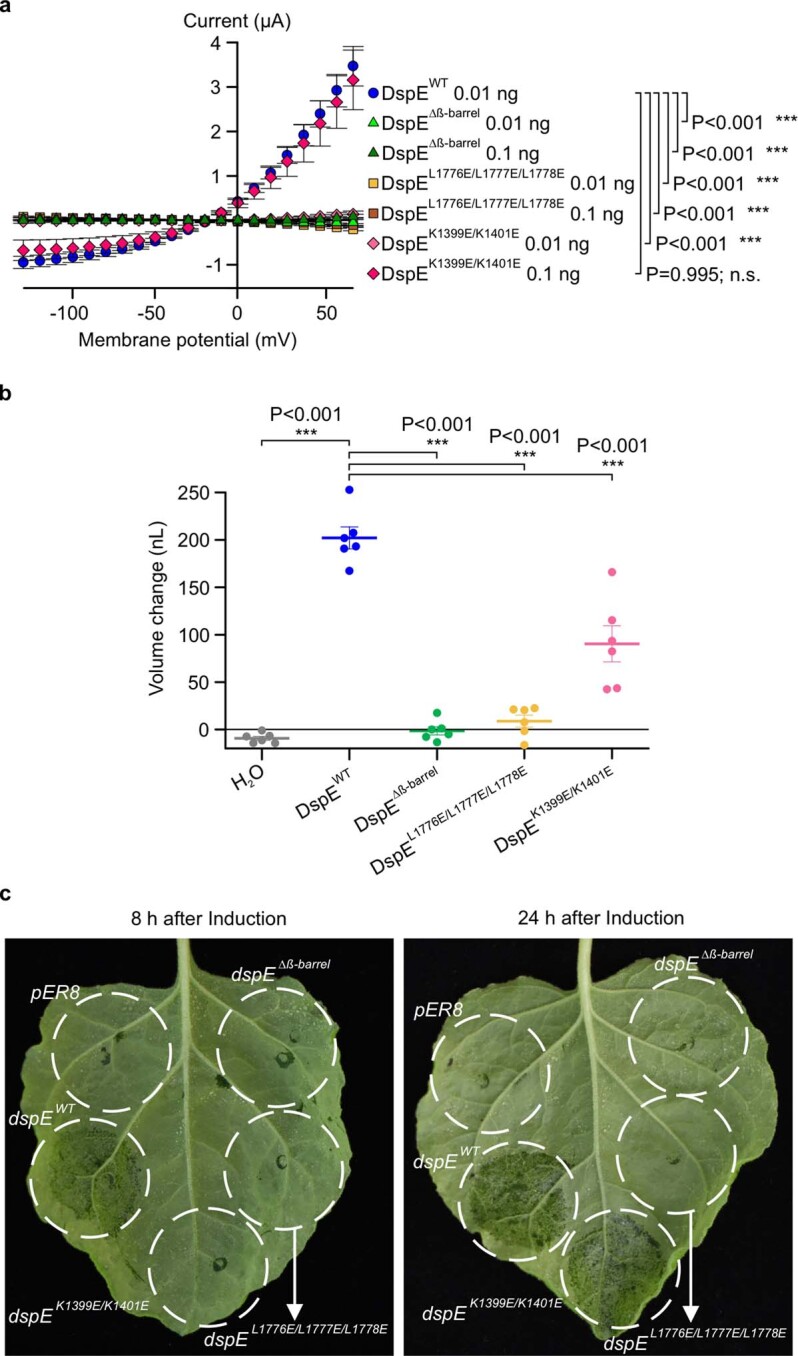
Functional analysis of mutant DspE proteins in Xenopus oocytes and tobacco. **a**, Effect of mutations on DspE-induced currents in oocytes. Mean ± s.e.m. (n = 5 oocytes) current values at different test pulses from oocytes expressing wild-type or mutant DspE proteins at 15 h after injection with 0.01 or 0.1 ng cRNA. Note: At 0.01 cRNA injection, all mutant DspE proteins did not induce currents. Next, 10-fold increase in mutant *dspE* cRNA (i.e., 0.1 ng) was injected into oocytes, revealing current induction by DspE^K1399E/K1401E^, suggesting the K1399E/K1401E double mutations only partially affect ion conductance, consistent with results in panels b and c. **b**, Effect of mutations on DspE-induced baseline swelling in oocytes. Plots represent mean ± s.e.m. (n = 6 oocytes) and individual values of increased oocyte volume in relation to its initial volume 15 h after 1 ng cRNA injection. **c**, Water-soaking assay in tobacco leaves. 1 × 10^8^ CFU/mL of *Agrobacterium tumefaciens* GV3101 containing pER8 empty vector, pER-*dspE*, pER-*dspE* mutants were syringe-inoculated into *Nicotiana benthamiana* leaves (infiltration areas circled) and kept at 22 °C for 24 h before leaves were painted with 90 µM estradiol to induce DspE expression. Water-soaking symptom (dark-coloured appearance) was assessed at 8 h and 24 h after estradiol induction, showing a complete loss of water-soaking induction by DspE^β-barrel^ and DspE^L1776E/L1777E/L1778E^, but only delayed water-soaking by DspE^K1399E/K1401E^. Experiments were independently performed two times. Two- (a) or one- (b) way ANOVA values and exact P-values for all comparisons are detailed in the Source Data files. See Extended Data Figs. 4b and d for immunoblotting of DspE and DspE mutant expression. Source data

We made several mutant derivatives of DspE to evaluate their functional consequences. As a negative control, DspE(Δβ-barrel) failed to induce water-soaking symptoms in *Nicotiana benthamiana* leaves, conduct ion currents, cause oocyte swelling or allow fluorescein release in the liposome assay (Fig. 2h and Extended Data Fig. 6). Similarly, triple substitution of three conserved hydrophobic, outward-facing residues (L1776, L1777 and L1778) of the predicted transmembrane region of DspE (location indicated by the orange asterisk in Fig. 1c) also abolished the DspE activities (Extended Data Fig. 6). Surface biotinylation of oocytes expressing DspE(Δβ-barrel) and DspE(L1776E/L1777E/L1778E) showed that they are no longer accessible by surface biotinylation and therefore probably cannot anchor across the membrane (Extended Data Fig. 4a). Finally, charge-reversal double substitution at K1399 and K1401, two inward-facing residues of the β-barrel (location indicated by the purple asterisk in Fig. 1d; not conserved among AvrE-family members), partially abolished the DspE activities (Extended Data Fig. 6). As a newly discovered family of channels, future research is needed to comprehensively define inward-facing residues that are critical for the function of DspE and other AvrE-family members.

## Inhibitors of DspE and AvrE channels

**Fig. 3:**
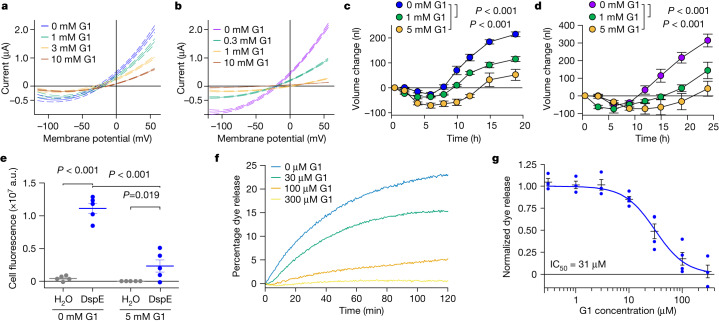
Inhibition of DspE and AvrE channels by the PAMAM dendrimer G1. The assays were carried out according to Fig. 2a,g, except with inhibitors added to the bath saline. **a**,**b**, DspE-dependent (**a**) and AvrE-dependent (**b**) currents were inhibited by G1. Solid lines represent fit values for current (µA) across the entire voltage range, with dashed lines representing the lower and upper 95% confidence interval of a quadratic polynomial regression for each treatment after subtracting control values. See Extended Data Fig. 4c for DspE expression in G1-containing ND96. **c**,**d**, Baseline swelling of oocytes injected with 1 ng *dspE* (**c**) or 20 ng *avrE* (**d**) cRNA was reduced in the presence of G1. Data show mean ± s.e.m. (*n* = 5 oocytes) increase in volume from the start point (at the time of injection) after subtracting control values. **e**, Inhibition of fluorescein uptake. G1 reduced fluorescein entry into oocytes expressing DspE as evaluated 6 h after injection with 1 ng *dspE* per oocyte. Data are presented as in Fig. 2e. **f**, DspE-mediated dye release from liposomes in the presence of increasing concentrations of G1. The result is representative of three experimental replicates. **g**, Dose-dependent inhibition of liposome dye release. IC_50_, half-maximal inhibitor concentration. Data show mean ± s.e.m. (*n* = 3 batches of liposome preparations for 0.3, 1, 3 and 300 µM G1; *n* = 4 batches of liposome preparations for 10, 30 and 100 µM G1). Experiments were independently carried out two times for **a**,**b**,**e**, four times for **c**,**d**, and seven times for **f**,**g**. *P* values were calculated using Two-way ANOVA (**e**) or two-way repeated measures ANOVA (**c**,**d**). Source data

**Extended Data Fig. 7:**
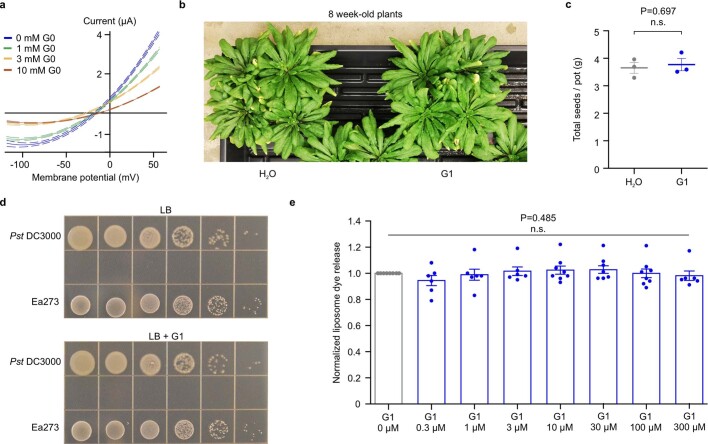
Effects of PAMAM inhibitors on DspE activities in oocytes and liposome and on plant phenotypes and bacterial growth. **a**, Inhibition of ion currents in oocytes. DspE-dependent currents were inhibited by G0 in a dose-dependent manner across all test pulses. Solid lines represent fit values for current (µA) across the entire voltage range, with dashed lines representing the lower and upper 95% confidence interval of a quadratic polynomial regression for each treatment after subtracting control values (n = 5 oocytes). **b**,**c**, PAMAM G1 does not affect plant growth or seed production of Arabidopsis Col-0 plant. Five-week-old Col-0 plants were sprayed with 10 µM PAMAM G1 every week until 12 weeks old. **b**, Picture of 8-week-old plants is shown here as an example. **c**, Seeds were collected at 15 weeks old. No obvious difference in plant appearance or seed production was observed. Data shown as mean ± s.e.m. (n = 3 pots of plants, each pot had 4 plants). **d**, PAMAM G1 does not impact bacteria multiplication in vitro (in LB agar medium). Ea273 or *Pst* DC3000 cells in the logarithmic growth phase were spotted, at a 10-fold serial dilution (from left to right), on low-salt LB agar plates with or without 50 μM PAMAM G1. Plates were kept at 28 °C for 2 days to visualize colonies. **e**, Normalized liposome dye release after addition of triton. Fluorescence readings of groups with increasing concentrations of G1 were normalized to the no compound controls and are shown as mean ± s.e.m. (n = 6 batches of liposome preparations for 0.3, 1, 3, and 300 µM G1; n = 8 batches of liposome preparations for 10, 30, and 100 µM G1). Experiments were performed two (a) or three (b,c,d,e) times with similar results. Two-tailed student’s *t*-test (c), One-way ANOVA on Ranks (e), and regression (a) values are detailed in the Source Data files. Source data

Next, we attempted to identify compounds of molecular diameters that could fit the predicted pores of AvrE/DspE channels and might therefore block AvrE/DspE activities. We focused on a class of synthetic PAMAM dendrimers, which have programmable molecular diameters^34^. For example, PAMAM G0 has a diameter of 15 Å, whereas PAMAM G1 has a diameter of 22 Å (https://www.dendritech.com/pamam.html). Notably, the currents passing through the DspE and AvrE channels were reduced by G1 in a dose-dependent manner, reaching 71% inhibition on the DspE channel and 93% inhibition on the AvrE channel, respectively, at 10 mM G1 at 50 mV test pulse (Fig. 3a,b), while not affecting DspE expression (Extended Data Fig. 4c). Similar inhibition by G0 was observed on oocytes expressing DspE, reaching 68% inhibition at 10 mM G0 at 50 mV test pulse (Extended Data Fig. 7a).

When G1 was added to the ND96 incubation buffer, the baseline swelling over time was also inhibited in a dose-dependent manner, reaching a maximum of 76% inhibition for 5 mM G1 at 19 h after injection (Fig. 3c). Again, a similar effect was observed when this inhibitor was tested on AvrE-expressing oocytes, with 89% inhibition for 5 mM G1 (Fig. 3d).

We also tested the effect of G1 on fluorescein uptake by oocytes expressing DspE, and found that it inhibited fluorescent dye uptake, reaching 79% inhibition by the end of the assay (Fig. 3e). Finally, we tested G1 on purified DspE protein reconstituted in liposomes using the DspE-dependent liposome dye release assay. We found that G1 dose-dependently inhibited the release of fluorescein from the soybean liposomes after the addition of DspE (Fig. 3f). Fitting of the dye release yielded an IC_50_ value of 31 µM for G1 (Fig. 3g). Together, these findings show that we have identified PAMAM G1 as an inhibitor of AvrE/DspE-family channels.

## PAMAM G1 inhibits bacterial infection

**Fig. 4:**
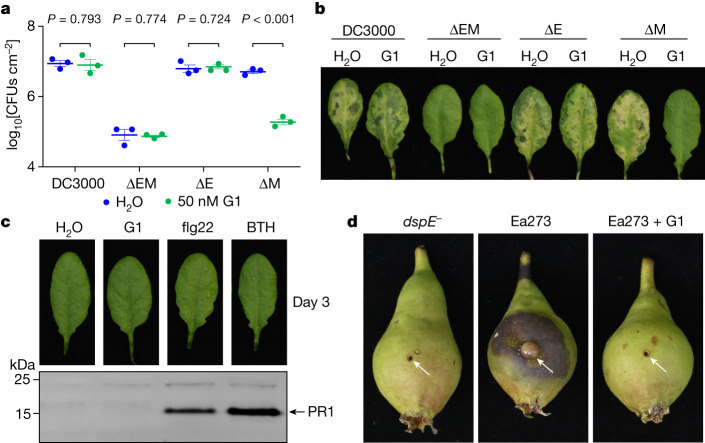
Effect of PAMAM G1 on bacterial infections. **a**,**b**, PAMAM G1 inhibits *Pst* DC3000 multiplication in an AvrE-dependent manner. A total of 1 × 10^6^ colony-forming units (CFUs) per millilitre of *Pst* DC3000, the *avrE*,*hopM1* double deletion mutant (ΔEM), the *avrE* single deletion mutant (ΔE) or the *hopM1* single deletion mutant (ΔM) were syringe-inoculated into leaves of *Arabidopsis* WT Col-0 plants, with or without 50 nM PAMAM G1. Populations of bacteria (mean ± s.e.m.; *n* = 3 leaf samples) (**a**) in leaves were determined at day 3 after infiltration. Disease symptom pictures (**b**) were taken at day 4 after infiltration. **c**, PAMAM G1 does not induce PR1 protein expression in *Arabidopsis*. *Arabidopsis* Col-0 leaves were syringe-infiltrated with 10 µM PAMAM G1. Plants were kept under high humidity (>95%) for 3 days at 23 °C. PR1 protein in leaves was detected by an anti-PR1 polyclonal antibody. As positive controls, 100 µM benzothiadiazole (BTH), a synthetic chemical analogue of salicylic acid, and 1 µM flg22, a synthetic peptide derived from the conserved N-terminal 22 amino acids of bacterial flagellin, induce PR1 expression. The uncropped gel image is shown in Supplementary Fig. 1g. **d**, PAMAM G1 inhibits fire blight disease by *E.* *amylovora* Ea273. Immature pear fruits were spot-inoculated (indicated by arrows) with 10 µl of 1 × 10^3^ CFUs ml^−1^ of Ea273 or the *dspE* mutant (*dspE*^−^), with or without 10 µM PAMAM G1. Inoculated pears were placed on a wet paper towel in a sterile box and incubated at 28 °C for 10 days. Diseased pears show areas with a dark, necrotic appearance. Experiments were carried out three times with similar results. *P* values were calculated using two-way ANOVA (**a**). Source data

The ability of PAMAM G1 to inhibit DspE and AvrE activities in oocytes and liposomes in vitro raised the possibility that we had identified a lead compound that could interfere with the AvrE and DspE virulence function in planta during bacterial infection. We first tested this possibility against the AvrE function during *P. syringae* infection. In many *P. syringae* strains, AvrE is functionally redundant to another effector, HopM1 (refs. ^11,13,19^). Mutation of either *avrE* or *hopM1* alone does not strongly affect *Pst* DC3000 virulence, but the *avrE*,*hopM1* double mutant is severely impaired in virulence^11,19^. Notably, whereas *avrE*-family effector genes are conserved widely^35^, *hopM1*-family genes and/or their secretion chaperone genes are subjected to natural genetic mutations as in the case of the major pandemic bacterial pathogen *P. syringae* pv. *actinidiae*^36^. We found that G1 effectively inhibited *Pst* DC3000 infection of *Arabidopsis* in an AvrE-dependent manner (that is, inhibition occurs in the *hopM1*-deletion mutant, which simulates natural mutations in the *hopM1* gene; Fig. 4a,b). Furthermore, inhibition of AvrE function by PAMAM G1 was not associated with induction of the PR1 protein, a marker for activation of salicylic acid-dependent immune responses in plants (Fig. 4c), or with negative effects on plant appearance (Extended Data Fig. 7b) or seed production (Extended Data Fig. 7c). Next we tested PAMAM G1 against *E.* *amylovora* infection. In *E.* *amylovora*, DspE plays an essential role in causing the devastating fire blight diseases, as the *dspA/E* mutant is largely nonpathogenic^7,8^. We found that G1 completely inhibited *E.* *amylovora* infection of highly susceptible pear fruits, phenocopying the *dspE* mutant of *E.* *amylovora*, an observation consistent with DspE being an indispensable virulence effector (Fig. 4d).

It is well known that type III secretion systems and their effectors are expressed and needed for bacterial growth only in host tissues, but not in vitro^1,2^. In accordance with this, G1 did not inhibit *E.* *amylovora* or *Pst* DC3000 growth in vitro (Extended Data Fig. 7d), providing further evidence that PAMAM G1 is truly a AvrE and DspE-specific virulence inhibitor, not just a nonspecific bactericidal antibiotic.

## Discussion

**Extended Data Fig. 8:**
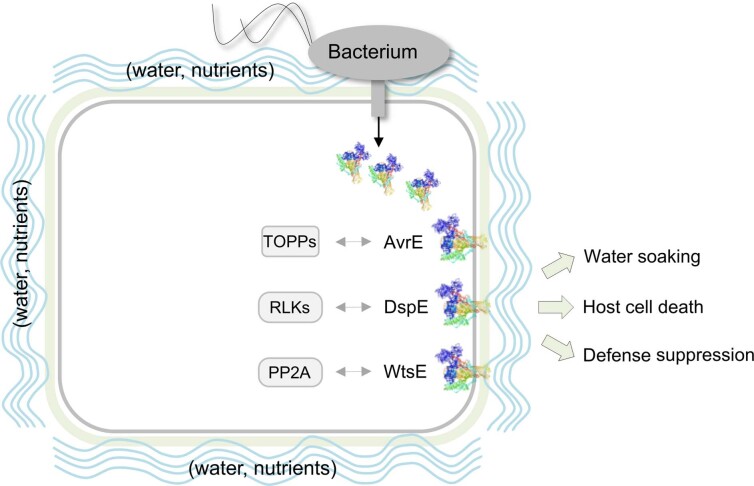
A working model for the molecular actions of AvrE-family effectors in plants. AvrE-family effectors act primarily as a novel class of water/solute-permeable channels dedicated to creating osmotic/water potential perturbation and a water/nutrient-rich apoplast, in which bacteria multiply within the infected plant tissues. AvrE-family effectors can additionally engage host proteins, including plant protein phosphatase PP2A subunits, type one protein phosphatases (TOPPs) and receptor-like kinases (RLKs), possibly to modulate AvrE-family channel properties or to optimize the pathogenic outcomes of AvrE/DspE-family channel activities, including water soaking, host cell death and defence suppression.

**Extended Data Fig. 9:**
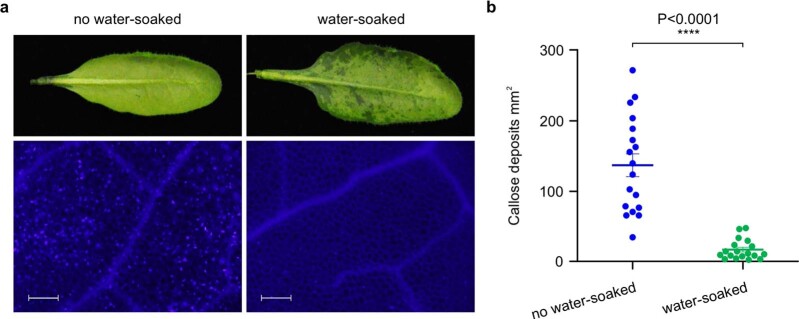
Water infiltration is sufficient to dampen callose deposition in Arabidopsis leaves. Arabidopsis Col-0 leaves were syringe-infiltrated with 1 µM flg22 and immediately covered by a clear plastic dome to maintain infiltrated leaves water-soaked (“water-soaked”; spotty, darker appearance) or air-dried to let infiltrated leaves returned to pre-infiltration appearance ( ~ 1 h) and then covered by a clear plastic dome (“no water soaked”; uniform, lighter appearance). **a**, Leaf pictures and callose (bright dots) images were taken at 8 h post flg22 infiltration. White scale bar indicates 100 µm. **b**, Quantification of callose deposition (mean ± s.e.m.; n = 18 leaf images [1 mm^2^ each] of callose deposit). Experiments were performed three times with similar results. Two-tailed student’s *t*-test values are detailed in the Source Data files. Source data

Since the initial report of AvrE in *P. syringae* almost three decades ago^37^, the central importance of AvrE-family effectors in diverse pathogenic bacterial species has attracted the attention of researchers. Before this study, however, researchers largely pursued a working hypothesis that, like most other type III effectors, AvrE/DspE-family effectors would target host proteins, RNA or DNA to exert their virulence functions. Our finding that AvrE-family effectors could directly function as water- and solute-permeable channels in this study is therefore striking and unexpected. We propose a new integrated model for the function of AvrE-family effectors (Extended Data Fig. 8). The carboxy-terminal halves of AvrE-family effectors act primarily as a new class of water- and solute-permeable channels dedicated to creating osmotic/water potential perturbation and a water- and nutrient-rich apoplast in which bacteria multiply within the infected plant tissues. Future research is needed to determine how AvrE-family channel activity, discovered in this study, mediates water, solute and nutrient flows and apoplast osmolarity in planta to generate macroscopic water soaking, host cell death and defence suppression in the infected tissue, as shown previously^6,13,19,25,38,39^. We found that infiltrating the *Arabidopsis* leaf apoplast with water (to simulate water soaking) was sufficient to suppress flg22-induced callose deposition (Extended Data Fig. 9a,b), suggesting that water soaking and suppression of certain immune responses (for example, defence-associated callose deposition) are linked processes.

As large proteins with potentially many protein-interacting interfaces, AvrE-family effectors can additionally engage host proteins, including plant protein phosphatase PP2A subunits, type one protein phosphatases and receptor-like kinases^22–25^, to affect aspects of AvrE/DspE functions. It is striking that the AvrE channel inhibitor PAMAM G1 can essentially phenocopy the *avrE* or *dspE* genetic mutations in abrogating all major virulence phenotypes associated with AvrE and DspE, including water soaking, tissue necrosis and bacterial multiplication in infected tissues (Fig. 4). Therefore, future research should examine the possibility that some of the identified AvrE, DspE- and WtsE-interacting host proteins may act through modulating AvrE-family channel properties and/or optimizing downstream pathogenic outcomes of AvrE-family channel activities.

In summary,  this study has unravelled the long-sought-after function of AvrE-family effectors. In addition, a chemical inhibitor of AvrE-family channels has been identified that appears to be broadly effective in reducing AvrE- and DspE-mediated bacterial infections. As such, the discovery of the water- and solute-permeable channel function of AvrE-family effectors has broad implications in the study of bacterial pathogenesis and bacterial disease control in plants.

## Methods

### Cloning, expression and purification of *E.amylovora* DspE *and* DspE(Δβ-barrel) in *Escherichia coli*

The *dspE* gene was PCR-amplified from the genomic DNA of *E.* *amylovora* strain Ea273 using the KOD hot start polymerase (Millipore Sigma) and the following primer set: DspE_Forward_primer: 5′-ATGGAATTAAAATCACTGGGAACTGAACACAAG -3′; DspE_Reverse_primer: 5′-GCTCTTCATTTCCAGCCCTTCCTTC-3′. The PCR product was cloned into a modified pET28a vector (Millipore Sigma) as a C-terminal fusion to maltose-binding protein (MBP) containing a His_8_ tag, a preScission protease cleavage site (PPX) and a Flag tag in the form of MBP–His_8_–PPX–Flag–DspE (hereinafter as MBP–DspE). Plasmid-transformed BL21(DE3) *E. coli* cells were grown in Luria–Bertani medium at 37 °C until the optical density at 600 nm (OD_600 nm_) reached 0.4–0.6, and were then induced with 0.1 mM IPTG and grown at 18 °C overnight. Collected cell pellets were resuspended in a lysis buffer containing 20 mM HEPES (pH 7.5), 300 mM NaCl and 2.5% glycerol, supplemented with cOmplete EDTA-free protease inhibitor tablet (Roche) and DNase I, and lysed by a French press. Following centrifugation at 20,000 r.p.m. for 30 min at 4 °C to remove cell debris, the fusion protein was purified using TALON Cobalt resin (Takara Bio). Following extensive washing in a buffer containing 20 mM HEPES (pH 7.5), 300 mM KCl, 2.5% glycerol, 1 mM ATP, 5 mM MgCl_2_ and 10 mM imidazole, the fusion protein was eluted in a buffer containing 20 mM HEPES (pH 7.5), 300 mM NaCl, 2.5% glycerol and 250 mM imidazole, and was further purified on a Superose 6 Increase 10/300 GL column (Cytiva Life Science) pre-equilibrated with a buffer containing 20 mM HEPES (pH 7.5), 150 mM NaCl and 1 mM dithiothreitol (DTT) at 4 °C. The protein fractions at the peak were aliquoted and flash-frozen at −80 °C for storage.

The construct of DspE(Δβ-barrel) (Δ1278–1566 + Δ1649–1813) was generated from the WT MBP–DspE construct described above through in-fusion cloning. The mutant protein was purified by following identical procedures as for the WT protein.

Representative size-exclusion chromatography profiles and SDS–polyacrylamide gel electrophoresis (PAGE) gels of the purified MBP–DspE and MBP–DspE(Δβ-barrel) proteins are shown in Extended Data Fig. 4g,h.

Constructs of MBP–DspE and MBP–DspE(Δβ-barrel) were made by the in-fusion cloning methods using the NEBuilder HiFi DNA assembly kit (New England Biolabs) with the following primer sets—WT DspE, *dspE*_forward_primer: 5′-GATGGAATTAAAATCACTGGGAACTGAACACAAG-3′; *dspE*_reverse_primer: 5′-GAAGGAAGGGCTGGAAATGAAGAGCTAATTGATTAA-3′; Vector_forward_primer: 5′-GAGCTAATTGATTAATACCTAGGCTGCTAAACAAAG-3′; Vector_reverse_primer: 5′-TTCTGTTCCAGGGGCCCGCGATGGAATTAAAATC-3′; DspE(Δβ-barrel) (Δ1278–1566 + Δ1649–1813), *dspE* forward_primer: 5′-CCTGGACAGTGCGGAGCCGGTGACCAGCAA-3′; *dspE* reverse_primer: 5′-AGGCTGCGGACAGCCACAGCGGAATAGCT-3′; Vector_forward_primer: 5′-CACAGCGGAATAGCTCAGGCTAATCCGCAG-3′; Vector_reverse_primer: 5′-GAATACGCTGTTGTCCCTGGACAGTGCGGA-3′.

### Cryo-EM sample preparation and data collection and processing

Cryo-EM grids were prepared using a Leica EM GP2 automatic plunge freezer in a humidity-controlled chamber operated at 10 °C and 85–90% relative humidity. Homemade gold Quantifoil R1.2/1.3 300-mesh grids were glow-discharged using the PELCO easiGlow glow discharge cleaning system (TED PELLA) before sample application. During sample freezing, a 3-μl sample of DspE (about 1.1 mg ml^−1^) was applied to freshly glow-discharged grids and incubated on grids for 60 s before blotting with Whatman #1 filter paper for 2.8 s. The grids were then immediately plunge-frozen in liquid ethane and stored in liquid nitrogen before data acquisition.

A total of 7,810 micrograph stacks were recorded on an FEI Titan Krios electron microscope (Thermo Fisher) operated at 300 kV equipped with a K3 direct electron detector (Gatan) operated in the counting mode. Micrograph stacks were collected at a nominal magnification of ×81,000 using a pixel size of 1.08 Å per pixel with a defocus range from −2.4 μm to −0.8 μm using the Latitude S (Version 3.51.3719.0, Gatan) automated image acquisition package. Each stack was exposed for 2.8 s with an exposure time of 0.047 s per frame, resulting in 60 frames per stack. The total dose was approximately 56.3 e^−^ Å^−2^ for each stack.

Motion correction and contrast transfer function (CTF) estimation were carried out with the patch motion correction model and patch CTF estimation module in cryoSPARC^40^. A total of 7,141 micrographs were selected from a total of 7,810 images on the basis of the CTF fitting resolution using a cutoff value of 4.0 Å. A total of about 2.4 million particles were picked using pre-trained TOPAZ^41^ models, of which about 167,000 particles corresponding to full-length protein with high-resolution features were selected to generate two-dimensional class averages. Cryo-EM samples of DspE showed a severe orientation bias of the particles, which prevented high-resolution reconstruction of the cryo-EM density maps.

### Cloning and in vitro transcription of *avrE* and *dspE* for oocyte experiments

The *avrE* or *dspE* open reading frame (ORF) was amplified with the following primer sets—*avrE* forward primer: 5′-TTGCCCGGGCGCCACCATGCAGTCACCATCGATCCACCGGA-3′ (Kozak sequence underlined); *avrE* reverse primer: 5′-CCTCTAGATTAGCTCTTCAGTTCGAACCCCTCT-3′; *dspE* forward primer: 5′-TTGCCCGGGCGCCACCATGGAATTAAAATCACTGGGAACTG-3′ (Kozak sequence underlined); *dspE* reverse primer: 5′-CCTCTAGATTAGCTCTTCATTTCCAGCCCTTCC-3′.

PCR-amplified *avrE* or *dspE* ORF (*Srf*I–*Xba*I fragment) was cloned into pGH19 (ref. ^42^) (digested with *Xma*I and *Xba*I) to create pGH-*avrE* or pGH-*dspE*. To prepare cRNA for oocyte injection, pGH-*avrE* or pGH-*dspE* was linearized with *Nhe*I, followed by in vitro transcription with T7 polymerase mMESSAGE mMACHINE Kit (Ambion).

For mutational analysis of DspE, point or deletion mutants of pGH-*dspE* were obtained using the Q5 Site-Directed Mutagenesis Kit (New England Biolabs) with the following primer sets—pGH-*dspE*^*Δβ-barrel*^ (that is, ∆1278–1566 + ∆1649–1813), ∆1278–1566 forward primer: 5′-GCGGAGCCGGTGACCAGCAACGATA-3′; ∆1278–1566 reverse primer: 5′-ACTGTCCAGGGACAACAGCGTATTC-3′; ∆1649–1813 forward primer: 5′-GGAATAGCTCAGGCTAATCCGCAGG-3′; ∆1649–1813 reverse primer: 5′-GCTGTGGCTGTCCGCAGCCTGTTGA-3′; pGH-*dspE*^*K1399E/K1401E*^, K1399E + K1401E forward primer: 5′-CTGGAGTTTGAGCTGACAGAGGATGAG-3′ (underline indicates mutation point); K1399E + K1401E reverse primer: 5′-GCTGTTTTGTAGCGTTCCTTGCAGGGT-3′; pGH-*dspE*^*L1776E/L1777E/L1778E*^, L1776E + L1777E + L1778E forward primer: 5′-GAGGAAGAGGGGACGAGCAACAGCCTG-3′ (underline indicates mutation point); L1776E + L1777E + L1778E reverse primer: 5′-CGCTGGGGTATTGAAGCCTTCGCTTTT-3′.

### *Xenopus laevis* oocyte preparation, injection and expression of DspE and AvrE

Oocytes were purchased as ovary from Xenopus1. The ovary was treated with 0.55 mg ml^−1^ collagenase B (0.191 U mg^−1^) in calcium-free ND96 saline^43^ (96 mM NaCl, 2 mM KCl, 1 mM MgCl_2_, 5 mM HEPES and 2.5 mM Na pyruvate, pH 7.5) for 20 min while on a nutating mixer at 21–22 °C. Immediately after treatment, the enzymatic solution was rinsed off the ovary with ND96 bath saline (96 mM NaCl, 2 mM KCl, 1 mM MgCl_2_, 1.8 mM CaCl_2_, 5 mM HEPES, 2.5 mM Na pyruvate and 0.5 mM theophylline, pH 7.5) several times, and cell clusters were spread on several 70-mm plastic culture dishes with ND96 bath saline for temporary storage in an 18 °C incubator. On the same day, the follicular cells and follicular membrane covering mature oocytes (stages IV and V) were manually peeled off with fine forceps, and oocytes were kept in ND96 bath saline at 18 °C until cRNA injection. cRNA was mixed with diethyl pyrocarbonate-treated water to defined concentrations necessary to deliver desired amounts (ranging from 0.01 ng to 20 ng) of cRNA per oocyte when injecting a volume of 27.6 nl. Injection was carried out with a nanoinjector (Nanoject II, Drummond Scientific) following the manufacturer’s directions. Diethyl pyrocarbonate-treated water was injected in control oocytes. Oocytes were kept in a 6-well plastic culture plate at 18 °C to allow expression of proteins. Incubation solution was either control (ND96 bath saline), ND96 with inhibitor (PAMAM G0 or G1, niflumic acid or fipronil), ND96 with 0.0005% fluorescein or ND96 with 0.1% GFP protein.

### Oocyte surface biotinylation assay

Oocytes were injected with 2 ng of WT or mutant *dspE* cRNA and incubated in bath ND96 saline for 15 h. Surface-exposed proteins were biotinylated and purified using the Pierce Cell Surface Protein Biotinylation and Isolation Kit (Thermo Fisher), following the manufacturer’s protocol with some modifications, as described previously^44^. Five oocytes were used per treatment (with biotin, without biotin or total cell extract). In brief, cells were rinsed three times in OR2 buffer^44^ before incubating in 2.5 ml of OR2 buffer for 10 min with or without sulfo-NHS-SS-biotin in 6-well culture plates in a benchtop orbital shaker, set at 85 r.p.m. at room temperature. Oocytes for the total cell extract treatment were immediately stored at −80 °C, whereas oocytes for biotinylation (with or without biotin) were rinsed three times in TRIS buffer^44^ before being placed in a 1.5-ml tube with 500 µl of lysis buffer containing 10 µl of Halt Protease Inhibitor Cocktail (Thermo Fisher). Lysis mix containing oocytes was homogenized by passing through a 20-gauge (G) needle 10 times, before incubation at 4 °C on a nutating mixer. The remaining steps followed the kit manufacturer’s protocol. Final samples were eluted with 200 µl elution buffer and mixed with 50 µl of 5× SDS sample buffer. In parallel, the five oocytes stored for total cell extract treatment were homogenized in 250 µl of 2× SDS sample buffer. For equal loading, 25 µl of total extract or avidin-pulldown biotinylated protein samples was added to each lane for SDS–PAGE.

### TEVC clamp recordings

Oocytes were injected with 0.01 ng of WT or mutant *dspE* cRNA or with 0.1 ng of *avrE* cRNA. AvrE seems less functional in oocytes than DspE. After about 15 h of incubation in bath ND96 saline (96 mM NaCl, 2 mM KCl, 1 mM MgCl_2_, 1.8 mM CaCl, 10 mM HEPES, pH 7.5), each oocyte was impaled by one voltage-sensing borosilicate microelectrode and one current-passing borosilicate microelectrode with a resistance of 0.5 ± 0.1 MOhm, while in 1 ml of an electrically grounded ND96 recording saline (96 mM NaCl, 2 mM KCl, 1 mM MgCl_2_, 1.8 mM CaCl, 10 mM HEPES, pH 7.5). Of note, in initial preliminary experiments, when oocytes were injected with a high amount of *dspE* or *avrE* cRNA (for example, 1 ng of *dspE* or 20 ng *avrE* cRNA per oocyte), membrane potentials at 24 h dropped close to 0 mV (Extended Data Table 1) and current conductance was very large (>50 µA), a condition at which the TEVC equipment no longer works properly. Thus, we lowered the cRNA input to 0.01 ng *dspE* per oocyte and 0.1 ng *avrE* per oocyte and evaluation time to 15 h after injection, which yielded a resting potential similar to that of oocytes injected with water control (Extended Data Table 1) and modest currents that TEVC was able to record.

For ion replacement experiments, variations of ND96 were prepared by replacing the major salt (that is, 96 mM NaCl) with 96 mM LiCl, KCl, RbCl, CsCl, choline-Cl, NDMG-Cl (*N*-methyl-d-glucamine hydrochloride), NaBr, NaI, NaClO_3_, NaBrO_3_ or Na-MES^45,46^. Currents were first recorded in ND96 recording buffer. To replace ND96 with a new cation or anion, 10 ml of a new ND96 solution was slowly added from one end of the recording chamber using a 10-ml plastic syringe with an 18-G needle, while the original ND96 was washed out using a vacuum outflow 20-G tube from the other end of the chamber. The glass microelectrodes were half-filled with 1.5% agar containing 3 M KCl. The electrodes were connected to an oocyte clamp amplifier (OC-725C, Warner Instrument) by chlorinated silver wires. The bath clamp headstage was connected to bath saline by two chlorinated silver wires inside a disposable polytetrafluoroethylene 18-G tubing filled with 1.5% agar containing 3 M KCl serving as agar bridges. The oocyte clamp amplifier was connected to a computer by an analog–digital interface (Digidata 1440A, Molecular Devices). The command voltage protocols and data acquisition were carried out in the pCLAMP v.10.7 software suite (Molecular Devices). Oocytes were clamped to a desired potential using the fast clamp mode with maximum clamp gain and current gain set to 0.1 V uA^−1^. Signal for both voltage and currents was recorded. After impaling an oocyte and before clamping it, both electrodes are capable of measuring the resting potential of that oocyte. Oocytes were clamped at their resting potential and test pulses of 100 ms towards more positive or more negative potentials in 10 mV increments were applied. The resultant current was recorded and analysed. Current amplitude was determined 10 to 20 ms after the start of the test pulse, the time at which there was the smallest or no overlaps with membrane capacitance or Ca-dependent Cl^−^ currents from endogenous oocyte channels^47^. As resting potential values across individual oocytes varied (probably owing to uncontrollable intrinsic differences in each oocyte, its size and in fine adjustment of electrode position and resistance), the voltage–current relationship data were fitted to a quadratic polynomial regression (SigmaPlot 12.5 Systat Software) providing intermediate values and 95% confidence intervals. This also allowed currents elicited by the test potentials on control oocytes to be subtracted from the currents in treatment oocytes, so the resultant values represent only DspE- or AvrE-mediated current flowing across the membrane. Comparison of the currents was carried out using a two-way ANOVA with Tukey’s test, with significance set to a *P* value < 0.05.

### Oocyte swelling assay

Oocytes injected with 1 or 2 ng *dspE* or 20 ng *avrE* cRNA per oocyte were imaged using Motic Images Plus 3.0 software connected to a Moticam X3 camera (Motic China) on an SHR Plan Apo 1×, working distance 60, magnification lens of a stereoscope (Nikon SMZ18). At 0.01 ng *dspE* or 0.1 ng *avrE* cRNA per oocyte that was used for TEVC recordings, no baseline oocyte swelling was observed. Baseline swelling began to be observed at >0.1 ng *dspE* or >10 ng *avrE* cRNA per oocyte. For baseline oocyte swelling, starting oocyte images were recorded immediately after each injection, and then every 2 h to 4 h for 24 h. Oocytes were kept in bath saline of 200 mOsm with or without PAMAM inhibitors in the stereoscope room at 18–19 °C for the entire period. Each picture depicting five oocytes (replicates) was analysed with Fiji v.2.3.0 software^48^. Data are presented as absolute volume at a given evaluation time or as change in volume in relation to the start point (immediately after cRNA injection). For hypoosmotic-induced swelling, oocytes expressing DspE or AvrE were transferred into a fivefold-diluted ND96 bath saline (40 mOsm) and were immediately imaged as described for baseline swelling once every 20 s for 10–20 min or until oocytes injected with *dspE* or *avrE* started to burst. Data are presented as change in volume in relation to the first picture in diluted saline. Pictures were also arranged in sequence to create time-lapse videos showing oocyte swelling and bursting. One-way ANOVA with Tukey’s test was used for multiple comparisons within a dataset, with significance set to a *P* value < 0.05. For dataset with repeated measures over time, as in the hypoosmotic-induced swelling assay, a two-way repeated measures ANOVA with Dunnett’s test was used instead, with significance also set to a *P* value < 0.05.

### Oocyte dye uptake assay

Two hours after injection with 1 or 2 ng of *dspE* or 20 ng of *avrE* cRNA, oocytes were placed in ND96 bath saline with or without 5 µg ml^−1^ fluorescein, 1 mg ml^−1^ GFP and/or 5 mM PAMAM G1 inhibitor and incubated until evaluation time, as indicated in the figure legends. Oocytes were rinsed twice in ND96 bath saline and imaged as described above for oocyte swelling assay, with a few exceptions: they were imaged at a ×2 magnification with either a bright-field or GFP-B filter. In the Motic Images Plus software, the green channel gain was increased to improve green fluorescence detection. Although specific values of the green channel gain value varied across different independent assays, all configurations were kept the same across all treatments within the same experiment. Bright-field and fluorescence images of each oocyte were stacked using Fiji software and the integrated density of fluorescence was measured within oocyte boundaries and subtracted from the fluorescence values observed on the image background, so data are presented as corrected total cell fluorescence (for short: cell fluorescence). Two-way ANOVA, with Tukey’s test, was used for multiple comparisons within a dataset, with significance set to a *P* value < 0.05.

### eGFP purification

For eGFP purification, pET28-*eGFP*^49^ was transformed into *E*. *coli* Rosetta(DE3). eGFP production was induced by adding 0.25 mM IPTG to bacterial culture for 4 h at 28 °C. eGFP was purified from total cell lysate using Ni-NTA agarose beads in the extraction buffer (50 mM Tris-Cl, pH 8.0, 250 mM NaCl, 5% glycerol, 0.1 mM phenylmethylsulfonyl fluoride). Before the oocyte uptake test, the buffer was exchanged to ND96 bath saline using Amicon Ultra-4 centrifugal filter units (MilliporeSigma).

### Western blot analysis

Five oocytes (15 mg) or 10 mg fresh plant leaf tissue was homogenized in 100 µl of 2× SDS sample buffer. After 10 min boiling, cell lysates were briefly centrifuged and 10 µl was loaded to each lane of an SDS–PAGE gel. After separation, proteins were blotted onto a PVDF membrane. AvrE, β-actin, DspE or PR1 was detected by anti-AvrE^20^ (1:5,000 dilution), anti-β-actin [HRP] (GenScript; 1:5,000), anti-DspE^50^ (1:5,000), anti-PR1 antibody (a gift from Xinnian Dong; 1:5,000), respectively, on an Invitrogen iBright 1500 system. The secondary antibodies anti-rabbit IgG (whole molecule)–alkaline phosphatase (Sigma) or anti-rabbit IgG (whole molecule)–HRP antibody (Sigma) were used with 1:10,000 or 1:5,000 dilution, respectively.

### Liposome preparation and liposome dye release assay

Soy extract lipids in chloroform were purchased from Avanti Polar Lipids and stored in glass vials^24^. These solutions were evaporated under a stream of nitrogen until a thin lipid film formed and then dried in a vacuum desiccator chamber overnight. On the second day, the lipid film was dissolved in a suspension buffer (HBS buffer: 20 mM HEPES, 300 mM NaCl, pH 8.0) containing 50 mM 5(6)-carboxyfluorescein (Novabiochem) or 50 mg ml^−1^ polysucrose 40–fluorescein isothiocyanate conjugate (FITC–polysucrose, molecular mass of 30–50 kDa with an estimated diameter of 80 Å, MilliporeSigma). To solubilize lipids, the solution in the glass vial was sonicated for 15 min and then incubated in a 37 °C water bath for at least 1 h. Then the lipid solution was subjected to eight freeze–thaw cycles, in which lipids were frozen in liquid nitrogen for 5 min and then thawed in a 37 °C water bath for 10 min, to reduce the formation of multilamellar liposomes. To control the liposome size, liposomes were extruded through a polycarbonate filter (200 nm, Whatman) 25 times using a mini extruder (Avanti Polar Lipids) with Hamilton glass syringes. Carboxyfluorescein– or FITC–polysucrose-loaded liposomes were purified by centrifugation at 41,000 r.p.m. for 20 min in a TLA 100.3 rotor incorporating three sequential wash steps. After the final wash, carboxyfluorescein– or FITC–polysucrose-loaded liposomes were resuspended in HBS buffer to give a final carboxyfluorescein-loaded liposome concentration of 1 mg ml^−1^ and FITC–polysucrose-loaded liposome concentration of 0.5 mg ml^−1^ (ref. ^51^).

Release of the liposome contents was assessed using the self-quenching property and fluorescence of carboxyfluorescein– and FITC–polysucrose. The HBS buffer composition in and outside the liposome was the same (20 mM HEPES, 300 mM NaCl, pH 8.0). Permeability induced by DspE was evaluated by incubating 10 µl DspE protein solution with 90 µl carboxyfluorescein- or FITC–polysucrose-loaded liposomes (0.25 μg μl^−1^). The fluorescence intensity was measured every 30 s continuously for 2 h after addition of the purified DspE protein (WT or mutant) to the liposomes in a SpectraMax M3 (Molecular Devices). Then 5 µl of 20% Triton X-100 (Sigma-Aldrich) was added to the 100-µl solution to fully release the dye and its readings were measured for 20 min. The average reading of the last 3 min was used for normalization (100% dye release). In the compound inhibition assays, the buffer, DspE protein and PAMAM G1 inhibitor, at a total volume of 10 µl, were first mixed thoroughly with pipetting, and then 90 µl carboxyfluorescein– or FITC–polysucrose-loaded liposomes was added to a total volume 100 μl. The spectrofluorometric excitation and emission parameters were set at the wavelengths of 485 and 510 nm for carboxyfluorescein– and FITC–polysucrose molecules.

The DspE protein stock solutions (2.5−25 μM) contained 1 mM DTT. The liposome assays were carried out at 0.05–0.15 μM DspE concentrations with the final DTT concentration less than 0.02 mM. The presence of DTT in the protein buffer did not affect the fluorescence of carboxyfluorescein (Extended Data Fig. 5e). Similarly, the presence of PAMAN G1 at concentrations in the range of 0.3–300 μM did not affect the intrinsic fluorescence of carboxyfluorescein (Extended Data Fig. 7e).

### Bacterial media and plant growth

Bacterial strains used were WT *Pst* strain DC3000 and its mutants: the *avrE* deletion mutant (ΔE)^11^, the *hopM1* deletion mutant (ΔM)^11^ and the *avrE* and *hopM1* deletion mutant (ΔEM)^11^ and WT *E.* *amylovora* strain Ea273 and its *dspE* mutant^8^. Bacteria were grown in low-salt Luria–Bertani medium at 28 °C. The antibiotic ampicillin, gentamicin, kanamycin, rifampicin or spectinomycin was added at 200, 10, 50, 100 or 50 µg ml^−1^, respectively. *Arabidopsis thaliana* Col-0 and Col-0/*DEX::his-avrE*^20^ plants were grown in Redi-Earth potting soil (Sun Gro Horticulture) in air-circulating growth chambers. Plants were grown at a relative humidity of 60%, temperature of 20 °C, light intensity of 100 µE m^−2^ s^−1^ and a photoperiod cycle of 8 h light–16 h dark. Four- to five-week-old plants were used for bacterial disease assay. Immature pear fruits were gifts from George Sundin at Michigan State University. *N. benthamiana* plants were grown in a growth chamber with 12 h light/12 h dark at 23 °C day and 21 °C night, about 55% humidity and about 100 μmol m^−2^ s^−1^ light intensity. Four- to six-week-old plants were used for transient expression assay.

### Bacterial disease assays

Disease assays with immature pear fruits were carried out as previously reported^8^. Pears were surface-sterilized with 10% bleach for 5 min and rinsed in sterile water twice. Then a small hole was made in the pear using a 200-µl tip. Ten microlitres of a solution containing 10^3^ CFUs per millilitre of Ea273 or the *dspE* mutant was loaded into the hole. Inoculated pears were placed on a wet paper towel in a sterile box to maintain high humidity at 28 °C for 10 days. Disease assays with *Arabidopsis* plants were carried out as follows. *Arabidopsis* plant leaves were infiltrated with *Pst* DC3000, ΔE, ΔM or ΔEM at 10^6^ CFUs per millilitre with a needle-less syringe. After signs of water soaking were no longer visible (within 1 h), plants were kept under high humidity (about 99%) at 23 °C. The population of bacteria in leaves was determined at day 3 post infiltration. Detached leaves were surface-sterilized in 75% ethanol for 30 s and rinsed in sterile water twice. Then, leaf discs (1 cm^2^ in diameter) were punched out and ground in 100 µl sterile water. Ten microlitres of each tenfold serial-diluted leaf extract was plated on Luria–Bertani rifampicin and kept at 28 °C for 24 h. CFUs were counted under a microscope before colonies started to coalesce and were analysed by GraphPad Prism software. Two-way ANOVA with Tukey’s test was used for multiple comparisons within a dataset, with significance set to a *P* value < 0.05. For inhibition assays, 50 nM PAMAM G1 was added to bacterial suspension and co-inoculated into plants.

### AvrE-family protein sequence alignments

Sequences of *E.* *amylovora* DspE, *P.* *carotovorum* DspE, *Pst* DC3000 AvrE and *P. stewartii* WtsE were aligned using Clustal Omega^52^. Sequences were from Uniprot (https://www.uniprot.org) as follows—*E. amylovora* Ea321 DspE (O54581), *P. carotovorum* Er18 DspE (D5GSK5), *Pst* DC3000 AvrE (Q887C9) and *P.* *stewartii* subsp. *stewartii* SS104 WtsE (Q9FCY7).

### Transient expression of DspE in *N. benthamiana*

*dspE* and *dspE* mutant ORFs were PCR-amplified with *dspE* ORF forward primer (5′-TTGGGCCCATGGAATTAAAATCACTGGGAACTG-3′, underline indicates *Apa*I site) and *dspE* ORF reverse primer (5′-TTTACTAGTTTAGCTCTTCATTTCCAGCCCTTCC-3′, underline indicates *Spe*I site) and pGH-*dspE* or pGH-*dspE* mutant plasmids as a template. PCR-amplified *dspE* and *dspE* mutant ORFs (*Apa*I–*Spe*I fragment) were cloned into the binary vector pER8^53^ to create pER-*dspE* and pER-*dspE* mutant constructs. All constructs were transformed into *Agrobacterium tumefaciens* GV3101 for the transient expression assay. A total of 1 × 10^8^ CFUs per millilitre of *A. tumefasciens* GV3101 containing pER8 empty vector, pER-*dspE* or pER-*dspE* mutant were syringe-inoculated into leaves of *N. benthamiana* and kept at 22 °C for 24 h before leaves were painted with 90 µM oestradiol. Eight hours later, leaf samples were collected for western blotting. Water-soaking and necrosis symptoms were recorded at 8 h and 24 h after oestradiol treatment under high humidity (>95%).

### *Arabidopsis* leaf protoplast swelling assay

Leaf mesophyll protoplasts were isolated from 5-week-old *Arabidopsis* Col-0 and transgenic Col-0/*DEX::his-avrE*^20^ following the tape sandwich method^54^. For the swelling test, isolated protoplasts were incubated in protoplast isolation medium (mannitol magnesium medium) containing 400 mM (isosmotic) or 320 mM (hypoosmotic) mannitol for 1 h. Protoplast images were taken using a Leica DM500 microscope with an ICC50W camera. Protoplast volumes were analysed with Image J v.1.53 software.

### Callose staining

Callose staining was carried out as described previously^13^. Callose images were taken using a Zeiss Axiophot D-7082 Photomicroscope. The number of callose depositions was determined with Quantity One 1-D analysis software v. 4.6.6 (Bio-Rad).

### Statistical analysis

Experimental sample size was chosen on the basis of previously published literature to be sufficient for statistical analyses. Three to four plants (biological replicates) per treatment and/or per genotype were analysed per individual experiment. Two or more independent experiments were carried out for all assays. The following statistical analyses were used: one-way ANOVA was used for multi-sample experiments with one variable, followed by Tukey’s honest significant difference test for multi-comparisons; two-way ANOVA was used for multi-variable analyses, followed by either Tukey’s honest significant difference test for multi-comparisons or Dunnett’s test for comparison against a common control treatment; two-way repeated measures ANOVA was used for repeated measures over the same experimental unit, followed by Dunnett’s test for comparison against a common control treatment; and Student’s *t*-test was used to compare two sets of data. If tests of normality of the residuals and equality of variances failed, the non-parametric alternatives ANOVA on ranks or the Mann–Whitney rank sum test were used instead. All statistical tests are described in the figure legends and Methods. Graphic plots were generated by SigmaPlot 12.5 and show mean ± s.e.m. and individual data points.

### Graphic design

Images and cartoons were created or assembled in CorelDRAW v.22 (Corel Corp. Ottawa, Canada) and PyMol (1.8.0.4). All graphics data were organized in Microsoft Excel v.2016 before being plotted on SigmaPlot 12.5 (Systat Software). Graphics were further edited for colour and arrangement as figure panels in CorelDRAW v.22 (Corel).

### Reporting summary

Further information on research design is available in the Nature Portfolio Reporting Summary linked to this article.

## Online content

Any methods, additional references, Nature Portfolio reporting summaries, source data, extended data, supplementary information, acknowledgements, peer review information; details of author contributions and competing interests; and statements of data and code availability are available at 10.1038/s41586-023-06531-5.

### Supplementary information

Supplementary Fig. 1 a–i, Whole-gel images for Extended Data Fig. 4a–f (a–f), Fig. 4c (g) and Extended Data Fig. 4g,h (h,i). Dashed rectangles show cropped areas.

Reporting Summary

Supplementary Data Source data for Extended Data Table 1.

Supplementary Video 1 AvrE rapid swelling and burst assay in *Xenopus* oocytes. After 20 ng of cRNA was injected and time was allowed for protein expression (24 h), oocytes were moved from a 200 mOsm saline solution to a 40 mOsm saline solution (5× dilution in ultrapure H_2_O). Note that AvrE-expressing oocytes further increase in size until burst mainly through the cRNA injection site where the extracellular matrix layer surrounding the plasma membrane is weaker than the rest of the oocyte. Cells were imaged every 20 s under a stereomicroscope (×7.5 magnification), pictures were assembled in order, and the final time-lapse video is at 3.33 Hz. Video was assembled in iMovie v10.3.4, Apple Inc.

Supplementary Video 2 DspE rapid swelling and burst assay in *Xenopus* oocytes. After 2 ng of cRNA was injected and time was allowed for protein expression (24 h), oocytes were moved from a 200 mOsm saline solution to a 40 mOsm saline solution (5× dilution in ultrapure H_2_O). Note that DspE-expressing oocytes further increase in size until burst mainly through the cRNA injection site where the extracellular matrix layer surrounding the plasma membrane is weaker than the rest of the oocyte. Cells were imaged every 20 s under a stereomicroscope (×7.5 magnification), pictures were assembled in order, and the final time-lapse video is at 3.33 Hz. Video was assembled in iMovie v10.3.4, Apple Inc.

### Source data

Source Data Fig. 2

Source Data Fig. 3

Source Data Fig. 4

Source Data Extended Data Fig. 2

Source Data Extended Data Fig. 3

Source Data Extended Data Fig. 4

Source Data Extended Data Fig. 5

Source Data Extended Data Fig. 6

Source Data Extended Data Fig. 7

Source Data Extended Data Fig. 9

## Figures and Tables

**Extended Data Table 1:**
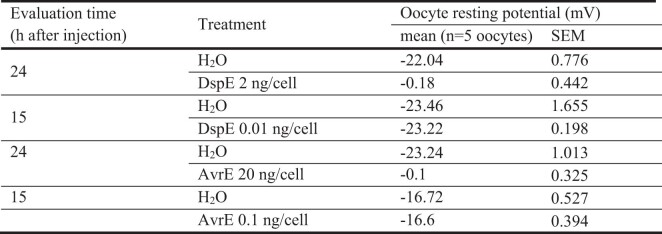
Effects of DspE and AvrE expression on oocyte membrane resting potential

Experiments were independently performed three times with similar results. Exact P-values for all comparisons are detailed in the Supplementary Data.

## Data Availability

Data needed to evaluate this paper are available in the main text and Supplementary Information. Uncropped gel and blot source data are provided in Supplementary Fig. 1. Gene and protein sequence data were obtained from Uniprot (https://www.uniprot.org) as follows: *E.* *amylovora* Ea321 DspE (O54581), *P.* *carotovorum* Er18 DspE (D5GSK5); *Pst* DC3000 AvrE (Q887C9), *P.* *stewartii* subsp. *stewartii* SS104 WtsE (Q9FCY7). Source data are provided with this paper.

## Code availability

No customized code was generated in this study. All bioinformatic tools and software used in this study are cited in the text.
