# Author Correction: Bacterial pathogens deliver water- and solute-permeable channels to plant cells

**DOI:** 10.1038/s41586-023-06995-5

**Published:** 2024-01-02

**Authors:** Kinya Nomura, Felipe Andreazza, Jie Cheng, Ke Dong, Pei Zhou, Sheng Yang He

**Affiliations:** 1 Department of Biology, Duke University, Durham, NC USA; 2 Howard Hughes Medical Institute, Duke University, Durham, NC USA; 3 Department of Biochemistry, Duke University School of Medicine, Durham, NC USA

**Keywords:** Bacterial pathogenesis, Effectors in plant pathology

Correction to: *Nature* 10.1038/s41586-023-06531-5 Published online 13 September 2023

In the version of the article originally published, the left panel of Extended Data Fig. 1d inadvertently displayed the AlphaFold2 model of *P. syringae pv. tomato* AvrE instead of *P. stewartii* WtsE. This panel has now been updated in the HTML and PDF versions of the article.

