# Supplementary material for: Bacterial pathogens deliver water- and solute-permeable channels to plant cells
Source: Nature. 2023 Sep 13;621(7979):586–91. doi: 10.1038/s41586-023-06531-5 (PMC10511319; doi:10.1038/s41586-023-06531-5)
Supplement: Supplementary file 1 — a–i, Whole-gel images for Extended Data Fig. 4a–f (a–f), Fig. 4c (g) and Extended Data Fig. 4g,h (h,i). Dashed rectangles show cropped areas. [file 41586_2023_6531_MOESM1_ESM.pdf]

nature portfolio
https://doi.org/10.1038/s41586-023-06531-5

Supplementary information

Bacterial pathogens deliver water- and solute-permeable channels to plant cells

In the format provided by the
authors and unedited

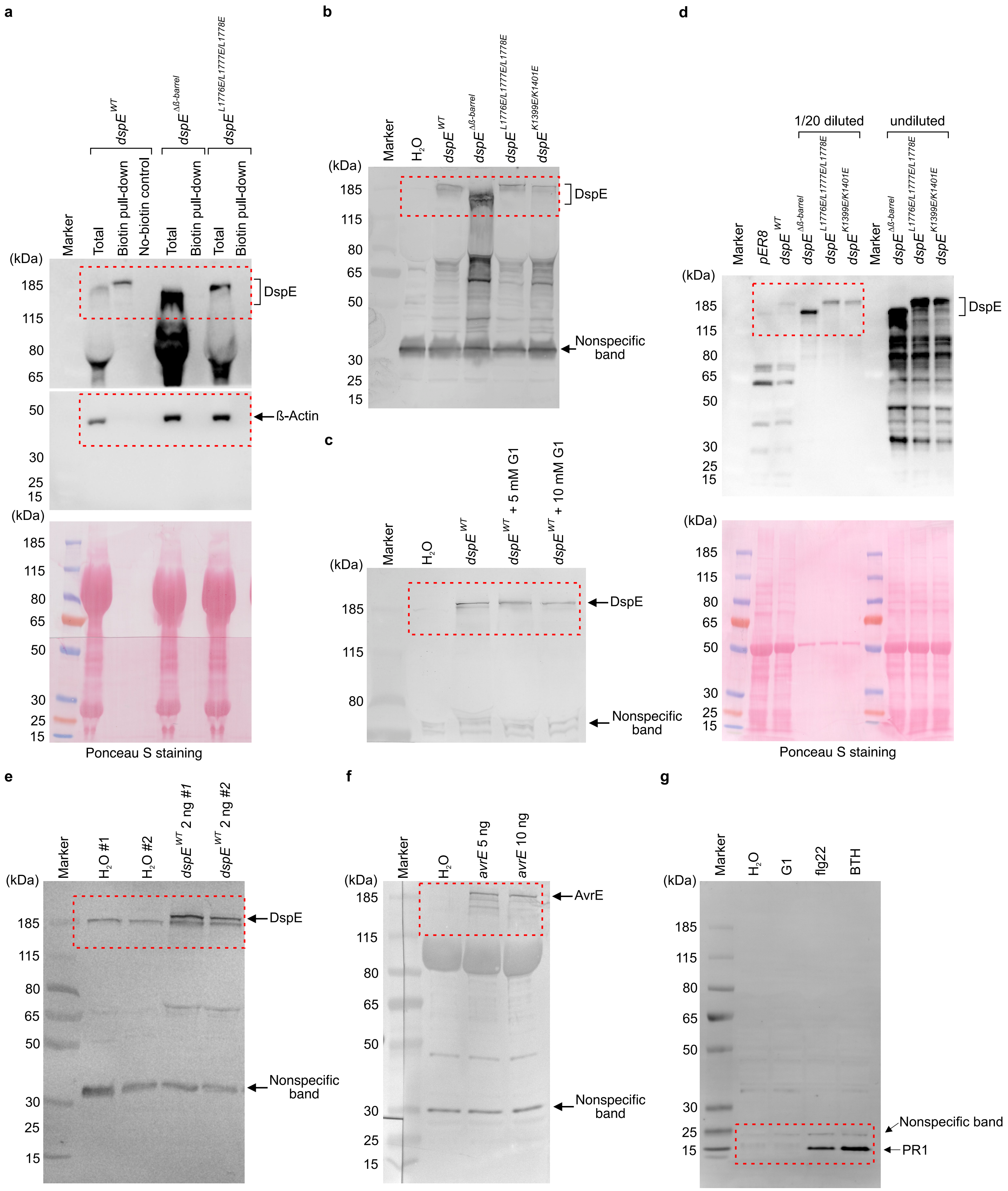
a
b
c
d
e
f
g
DspE
ß-Actin
Nonspecific band
AvrE
PR1
Marker
Total
Biotin pull-down
No-biotin control
H2O
G1
flg22
BTH
1/20 diluted
undiluted
(kDa)
Ponceau S staining

h

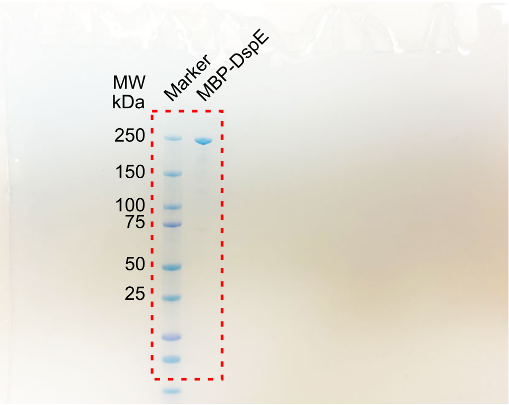

MW
kDa
Marker
MBP-DspE
250
150
100
75
50
25

I

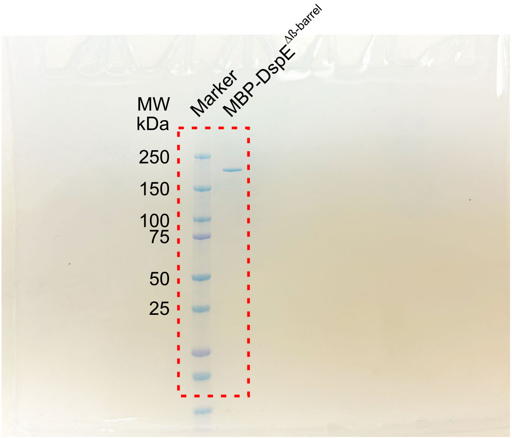

MW
kDa
Marker
MBP-DspE$^{\Delta\beta\text{-barrel}}$
250
150
100
75
50
25
