# Supplementary material for: Bacterial pathogens deliver water- and solute-permeable channels to plant cells
Source: Nature. 2023 Sep 13;621(7979):586–91. doi: 10.1038/s41586-023-06531-5 (PMC10511319; doi:10.1038/s41586-023-06531-5)
Supplement: Supplementary file 2 — Reporting Summary [file 41586_2023_6531_MOESM2_ESM.pdf]

nature portfolio

Corresponding author(s): Sheng Yang He, Pei Zhou, Ke Dong

Last updated by author(s): 07/20/2023

# Reporting Summary

Nature Portfolio wishes to improve the reproducibility of the work that we publish. This form provides structure for consistency and transparency in reporting. For further information on Nature Portfolio policies, see our Editorial Policies and the Editorial Policy Checklist.

## Statistics

For all statistical analyses, confirm that the following items are present in the figure legend, table legend, main text, or Methods section.

| n/a | Confirmed | |
|---|---|---|
| ☐ | ☒ | The exact sample size (*n*) for each experimental group/condition, given as a discrete number and unit of measurement |
| ☐ | ☒ | A statement on whether measurements were taken from distinct samples or whether the same sample was measured repeatedly |
| ☐ | ☒ | The statistical test(s) used AND whether they are one- or two-sided<br>*Only common tests should be described solely by name; describe more complex techniques in the Methods section.* |
| ☐ | ☒ | A description of all covariates tested |
| ☐ | ☒ | A description of any assumptions or corrections, such as tests of normality and adjustment for multiple comparisons |
| ☐ | ☒ | A full description of the statistical parameters including central tendency (e.g. means) or other basic estimates (e.g. regression coefficient) AND variation (e.g. standard deviation) or associated estimates of uncertainty (e.g. confidence intervals) |
| ☐ | ☒ | For null hypothesis testing, the test statistic (e.g. *F*, *t*, *r*) with confidence intervals, effect sizes, degrees of freedom and *P* value noted<br>*Give P values as exact values whenever suitable.* |
| ☒ | ☐ | For Bayesian analysis, information on the choice of priors and Markov chain Monte Carlo settings |
| ☒ | ☐ | For hierarchical and complex designs, identification of the appropriate level for tests and full reporting of outcomes |
| ☒ | ☐ | Estimates of effect sizes (e.g. Cohen's *d*, Pearson's *r*), indicating how they were calculated |

*Our web collection on statistics for biologists contains articles on many of the points above.*

## Software and code

Policy information about availability of computer code

| | |
|---|---|
| Data collection | Callose deposition: Zeiss Axiophot D-7082 Photomicroscope<br>Western blots: Invitrogen iBright 1500 system<br>Two Electrode Voltage Clamp: pCLAMP v.10.7 software suite<br>Oocyte Swelling and Dye uptake: Motic Images Plus 3.0<br>Liposome dye release assay: Molecular Devices SpectraMax M3<br>Cryo-EM data were collected using the Latitude S (Version 3.51.3719.0) on ThermoFisher Krios G3i Cryo TEM.<br>Protoplast swelling: Leica DM500 microscope with ICC50W camera |
| Data analysis | Callose deposition: Quantity One 1-D analysis software v. 4.6.6 (Bio-Rad)<br>Statistics and graph production: GraphPad Prism 9 software, SigmaPlot 12.5<br>Two Electrode Voltage Clamp: pCLAMP v.10.7 software suite, SigmaPlot 12.5<br>Oocyte Sweeling and Dye uptake: Fiji v2.3.0 (ImageJ2) software, SigmaPlot 12.5<br>Cryo-EM data analysis: cryoSPARC version 4.0.2, Topaz version 0.2.3<br>Inhibition of liposome dye release assay: Excel version 2016.<br>AlphaFold Model: AlphaFold Colab implementation of AlphaFold v2.3.0.<br>Protoplast Sweeling: ImageJ1.53 software |

For manuscripts utilizing custom algorithms or software that are central to the research but not yet described in published literature, software must be made available to editors and reviewers. We strongly encourage code deposition in a community repository (e.g. GitHub). See the Nature Portfolio guidelines for submitting code & software for further information.

# Data

Policy information about availability of data

All manuscripts must include a data availability statement. This statement should provide the following information, where applicable:

- Accession codes, unique identifiers, or web links for publicly available datasets
- A description of any restrictions on data availability
- For clinical datasets or third party data, please ensure that the statement adheres to our policy

Data needed to evaluate this paper is available in the main text and Supplementary Information. Uncropped gel and blot source data are provided in Supplementary Figures. Source data (with statistical analyses) for Figs. 1–4, Extended Data Table 1, and Extended Data Figures 1-9 are provided with this paper. Gene and protein sequence data were obtained from uniprot (https://www.uniprot.org) as following: Erwinia amylovora Ea321 DspE: https://www.uniprot.org/uniprotkb/O54581/entry; Pectobacterium carotovorum Er18 DspE: https://www.uniprot.org/uniprotkb/D5GSK5/entry; Pseudomonas syringae pv. tomato DC3000 AvrE: https://www.uniprot.org/uniprotkb/Q887C9/entry; Pantoea stewartii subsp. stewartii SS104 WtsE: https://www.uniprot.org/uniprotkb/Q9FCY7/entry.

# Human research participants

Policy information about studies involving human research participants and Sex and Gender in Research.

| | |
|---|---|
| Reporting on sex and gender | N/A |
| Population characteristics | N/A |
| Recruitment | N/A |
| Ethics oversight | N/A |

Note that full information on the approval of the study protocol must also be provided in the manuscript.

# Field-specific reporting

Please select the one below that is the best fit for your research. If you are not sure, read the appropriate sections before making your selection.

☒ Life sciences ☐ Behavioural & social sciences ☐ Ecological, evolutionary & environmental sciences

For a reference copy of the document with all sections, see nature.com/documents/nr-reporting-summary-flat.pdf

# Life sciences study design

All studies must disclose on these points even when the disclosure is negative.

| | |
|---|---|
| Sample size | Sample size and statistical analyses are described in the relevant figure legends. Sample size was determined based on experimental trials and with consideration of previous publications (e.g., bacterial quantification assay: PMID: 35247331, https://www.nature.com/articles/nature20166/; oocyte assay: PMID: 23821746, https://doi.org/10.1073/pnas.1305118110; liposome assay: PMID: 17574688, https://pubmed.ncbi.nlm.nih.gov/17574688/) on similar experiments to allow for confident statistical analyses. There were no statistical methods used to predetermine sample sizes. |
| Data exclusions | No data that pass quality control were excluded from statistical analysis. |
| Replication | The number of independent replication for each experiment is described in the relevant figure legends. Two or more independent experiments were performed for all assays. Results were ensured to be reproducible in all repeats with the same trend. |
| Randomization | Plants were grown side-by-side randomly in environmentally-controlled growth chambers (light, temperature, humidity) to control other covariates and to minimized unexpected environmental variations. Leaf samples of similar age were collected from plants at the indicated ages and assessed randomly. Xenopus oocytes assays were randomized. Healthy oocytes were chosen, and then randomly split to be injected with each of the treatments. Oocytes injected with same treatment were then randomly split into each of several bath saline treatments, when applicable. All oocytes/treatments were distributed randomly in each well of six well culture plates, kept at the same temperature condition. Evaluation for treatments was done randomly. Replicates (individual oocyte cells) of each treatment were imaged at once in a single picture. |
| Blinding | Researchers were not blinded to allocation during plant and oocyte experiments and outcome assessment. This is in part because plants and oocytes with different treatments can exhibit phenotypes that make them identifiable visually. Thus, blinding was not possible in these cases. Routine practices included more than one author observing/assessing phenotypes, whenever possible. |

# Reporting for specific materials, systems and methods

We require information from authors about some types of materials, experimental systems and methods used in many studies. Here, indicate whether each material, system or method listed is relevant to your study. If you are not sure if a list item applies to your research, read the appropriate section before selecting a response.

## Materials & experimental systems

| n/a | Involved in the study |
|---|---|
| ☐ | ☒ Antibodies |
| ☒ | ☐ Eukaryotic cell lines |
| ☒ | ☐ Palaeontology and archaeology |
| ☒ | ☐ Animals and other organisms |
| ☒ | ☐ Clinical data |
| ☒ | ☐ Dual use research of concern |

## Methods

| n/a | Involved in the study |
|---|---|
| ☒ | ☐ ChIP-seq |
| ☒ | ☐ Flow cytometry |
| ☒ | ☐ MRI-based neuroimaging |

## Antibodies

| | |
|---|---|
| Antibodies used | Anti-AvrE antibody produced in rabbit (custom made; see validation publication below)<br>Anti-beta Actin [HRP] antibody produced in mouse, GenScript, Cat. No. A00730, Lot No. 20A002083<br>Anti-DspE antibody produced in rabbit (custom made; see validation publication below)<br>Anti-PR1 antibody produced in rabbit (custom made; see validation publication below)<br>Anti-Rabbit IgG (whole molecule)–Alkaline Phosphatase antibody produced in goat, Sigma, Cat. No. A3687, Lot No. SLBV4176<br>Anti-Rabbit IgG (whole molecule)–HRP antibody produced in donkey, Sigma, Cat. No. GENA934, Lot No. 17271476 |
| Validation | Anti-AvrE antibody: PMID: 26206852, https://www.ncbi.nlm.nih.gov/pmc/articles/PMC4577396/<br>Anti-beta Actin antibody: https://www.genscript.com/antibody/A00730-THE_beta_Actin_Antibody_HRP_mAb_Mouse.html<br>Anti-DspE antibody: PMID: 11401717, https://onlinelibrary.wiley.com/doi/10.1046/j.1365-2958.2001.02455.x<br>Anti-PR1 antibody: PMID: 15890886, https://pubmed.ncbi.nlm.nih.gov/15890886/<br>Anti-Rabbit IgG–Alkaline Phosphatase antibody: Sigma A3687, https://www.sigmaaldrich.com/US/en/product/sigma/a3687<br>Anti-Rabbit IgG–HRP antibody: https://www.sigmaaldrich.com/US/en/product/sigma/gena9341ml |
